# Vector venom: venomics of *Aedes albopictus* reveals a large enzyme repertoire and novel cecropins with activity against *E. coli*

**DOI:** 10.1038/s44386-026-00041-w

**Published:** 2026-03-02

**Authors:** Ludwig Dersch, Jonas Krämer, Sabine Hurka, Maik Damm, Ole Bohlken, Alejandra Centurión, Bodunrin Omokungbe, Lennart Schulte, Michael Marner, Kornelia Hardes, Till F. Schäberle, Andreas Vilcinskas, Tim Lüddecke

**Affiliations:** 1 Animal Venomics Lab, Fraunhofer Institute for Molecular Biology and Applied Ecology, Giessen, Germany; 2 LOEWE-Centre for Translational Biodiversity Genomics, Frankfurt a. M., Germany; 3 Institute for Insect Biotechnology, Justus-Liebig-University of Giessen, Giessen, Germany; 4 Fraunhofer Institute for Molecular Biology and Applied Ecology, Branch for Bioresources, Giessen, Germany; 5 BMFTR Junior Research Group in Bioeconomy (BioKreativ) “SymBioÖkonomie”, Fraunhofer Institute for Molecular Biology and Applied Ecology, Giessen, Germany; 6 BMFTR Junior Research Group in Infection Research “ASCRIBE”, Fraunhofer Institute for Molecular Biology and Applied Ecology, Gießen, Germany

**Keywords:** Biochemistry, Biotechnology, Microbiology

## Abstract

Mosquitoes are vectors of deadly diseases and pose a global health threat. Particularly, the Asian tiger mosquito *Aedes albopictus* can transmit several pathogens, and is expanding into temperate regions. During blood feeding, mosquitoes inject chemically complex saliva, here referred to as venom, which modulates hemostasis, inflammation, immune response and pathogen transmission. In-depth knowledge of mosquito venom is crucial for understanding disease biology and enabling biodiscovery. We present a venomics study of *Ae. albopictus* and identify 119 distinct proteins validated by mass spectrometry and transcriptomics. The venom is rich in enzymes (e.g., hydrolases and Apyrases) and non-enzymatic components (e.g., odorant binding proteins and protease inhibitors). Additionally, we identified six novel cecropin family antimicrobial peptides. Structural analyses indicate an amphipathic N-terminus, hinge region, and hydrophobic C-terminus consistent with type II channel formation. Functional assays revealed that these cecropins exert potent effects on *E. coli* while leaving mammalian epithelial cells and erythrocytes unaffected. Overall, our study reveals that mosquito venom is a source of diverse biomacromolecules, deepening our understanding of its physiology, vector biology, and biochemical ecology. This opens paths for new mosquito-control strategies and drug discovery.

## Introduction

Mosquitoes are considered the world’s deadliest animals due to their capacity to transmit an array of pathogens that cause severe infectious diseases^1^. Vector-borne diseases account for more than 17% of all infectious diseases globally and are responsible for more than 700,000 deaths annually. For instance, malaria alone leads to approximately 249 million cases and over 608,000 deaths each year^2^. Dengue, the most prevalent mosquito-borne viral infection, threatens more than 3.9 billion people in 139 countries and is associated with 96 million symptomatic cases and an estimated 40,000 deaths annually^2^. An important vector species of this disease is the Asian tiger mosquito *Aedes albopictus*. It is capable of transmitting up to 34 arboviruses and can enhance nematode infection^3–8^. These mosquito-borne diseases are often regarded as tropical, but due to human-mediated dispersal and climate change, the range of *Aedes albopictus* now extends beyond the tropics into temperate regions^9^.

As hematophagous Diptera, adult female mosquitoes inject a complex cocktail of pharmacologically relevant bioactive components into their hosts to facilitate blood meals^10–12^. These factors not only assist in overcoming host defenses but also interact synergistically with pathogens, having direct repercussions on their virulence^13^. Traditionally, the secretions injected during blood meal are considered salivary secretions, and the totality of its chemical arsenal is referred to as “sialome”^14,15^. Conceptually, the blood meal-facilitating secretions of mosquitoes and other hematophagous Diptera also align with the formal definition of venom sensu Fry et al. 2019: “ …a secretion, produced in a specialized gland in one animal, and delivered to a target animal through the infliction of a wound (regardless of how tiny it) a venom must further contain molecules that disrupt normal physiological or biochemical processes so as to facilitate feeding or defense by the producing animal”^14^, as well as others^16–18^. This perspective emphasizes the impact such secretions can have on physiological processes and recognizes the potentially greater diversity of bioactive compounds. Therefore, modern works acknowledge the venomous nature of mosquitoes and consider their chemical venom arsenal to be an important yet neglected subject^18,19^. For the purpose of this study, we hereafter refer to the salivary secretions of mosquitoes as venom. The venom of mosquitoes is a decisive factor in the complex triad of vector, pathogen, and host interactions where it serves as a mediating agent. On one hand, it is the active principle that facilitates blood meals and, partially, influences pathogen virulence. On the other hand, it contains several potent biomolecules that target the host immune and cardiovascular systems^20^. The injected venom can increase vascular permeability and manipulate immune cell recruitment. Within the salivary glands it creates an environment promoting pathogen survival and replication^13^. Hence, improving our understanding of mosquito venom composition, bioactivity, and molecular mechanisms can offer valuable insights for developing vaccines, novel insecticides and exploring potential vector control strategies, such as gene silencing via RNA interference^13,21,22^. For example, a recent study identified a labrum-interacting protein in *Ae. albopictus* venom that promotes the penetration of host skin by activating mouthpart movements and salivation. These proteins represent promising targets for disrupting blood feeding and thus reducing disease transmission^23^. Likewise, other previously identified mosquito venom components, such as peptides from the cecropin family, have shown antimicrobial activity^24,25^. These molecules may hold therapeutic potential or serve as lead compounds for novel antimicrobial agents^26,27^.

The methodological restraints of analytical and preparative chemistry have prevented the efficient analysis of venoms and their components from small invertebrates, such as mosquitoes^28–30^. Only recently, through the application of modern systems biology methods paired with biotechnology approaches, the targeted and efficient disentanglement of such venom systems has become feasible and has given rise to a new research field referred to as modern venomics^29^. Although these methods have paved the way for theoretical analysis of venoms from the smallest venomous lineages, including various insects, arachnids, and crustaceans, only a handful of these species have been investigated thus far^19,30^. Much remains to be learned about the chemical arsenals of the many remaining lineages, which include mosquitoes^18^. With the importance of providing a better understanding of vector biology and venom biodiscovery, an in-depth analysis of mosquito venoms via modern venomics appears particularly promising. In this study, we aimed to broaden the understanding of the molecular composition, function, and translational potential of mosquito venoms by studying the venom system of the invasive Asian tiger mosquito *Ae. albopictus*. This approach allows us to elucidate mosquito venom from different perspectives, as provided by traditional sialotranscriptomic methods. We offer new insights into the molecular mechanisms of blood feeding and vector competence and open up new translational avenues for mosquito venom studies.

## Results

### A transcriptomic perspective on the *Ae. albopictus* venom system

For a first assessment of the *Ae. albopictus* venom system, we analyzed the salivary gland transcriptome. Therefore, we dissected salivary glands from the thorax of 60 adult female specimens. The salivary gland samples were pooled, and mRNA was extracted for subsequent transcriptome sequencing. Sequencing revealed 19,201,370 paired-end reads (total of 5.8 Gbp, GC content 43.9%, Q30 88.0%), 13,278,893 of which remained after quality control. The concatenated transcriptome assembly revealed 346,630 contigs, to which our trimmed reads were mapped at a rate of 95.89%. A total of 4,555,524 open reading frames (ORFs) were identified and extracted. Following additional processing, we determined 2374 ORFs with an assigned InterPro ID and a predicted signal peptide, accounting for a total of 2,699,363 TPM. This ORF list served as an input database for our proteome analysis. The assigned ORFs most likely represent precursors of secreted polypeptides, a pivotal feature of venom components, and were thus further analyzed. The molecular weights ranged from 2.3 kDa to 369.3 kDa, with sequence lengths of 21–3344 amino acids (Supplementary data).

**Fig. 1:**
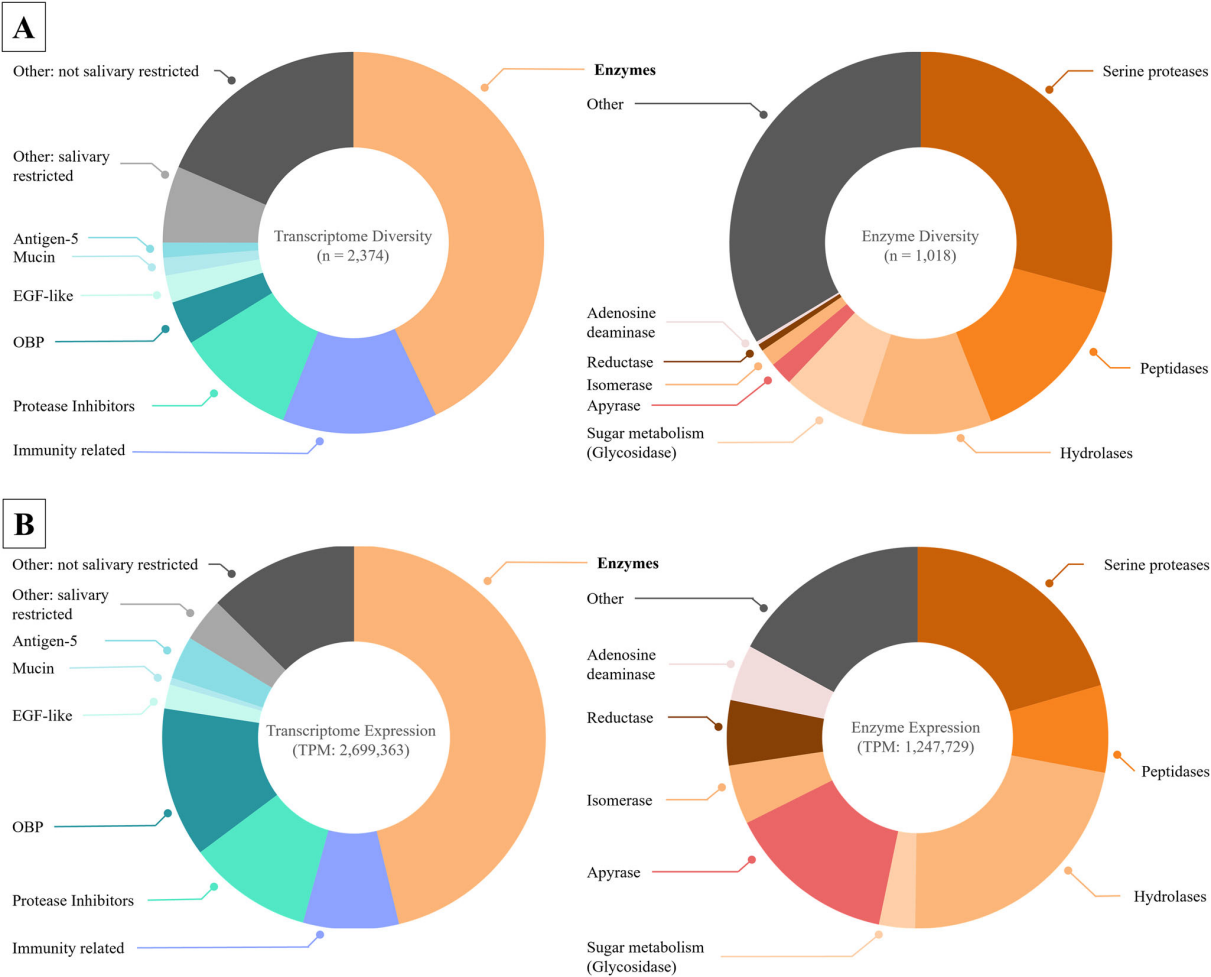
*Aedes albopictus* salivary gland transcriptome by protein family. **A** Total number of unique transcripts for each protein family. The left panel displays the whole transcriptome diversity consisting of 2374 transcripts. The right panel focuses on enzymes and their subfamilies, with a total of 1018 transcripts. **B** Relative expression levels (TPM scores) summed for each protein family. The left panel shows the whole transcriptome expression profile, which included the expression of seven families and others, for a total of 2,699,363 TPM. The enzyme expression data are given in the right panel, considering eight enzyme families and others, for a total of 1,247,729 TPM.

First, we explored the diversity of the whole transcriptome (Fig. 1A). Compounds annotated as enzymes appeared to be the most diverse class, representing 43% of all transcripts. Immunity-related peptides were the second most diverse class with 13%. This group contained several components that were similar to defensins, attacins, and cecropins. Next, 10% of the transcripts were protease inhibitors, and odorant-binding proteins (OBP), mainly D7 family, accounted for 4% of the transcripts. Mucins and epidermal growth factor (EGF)-like transcripts were detected with only 2% each, while Antigen-5 transcripts (CAP family) were represented in only 1%. A total of 6% of all transcripts could not be defined to specific classes and are here classified as “Other: salivary restricted”; these transcripts may feature protein families with roles for their venom or the salivary gland. The remaining 18% of the transcripts were similar to those of various housekeeping proteins and were classified as “Other: not salivary restricted”. As putative enzymes dominate the venom composition of *Ae. albopictus*, we investigated this class in more detail (Fig. 1A, right panel). Within the enzyme class, serine proteases are the most diverse and feature 29% of all enzymes identified. Other peptidases represented 15% of the enzymes. Hydrolases were found to represent 11% of the enzyme transcripts. Various Glycosidases and transcripts potentially relevant for sugar metabolism were detected with 7%. Apyrases display 2% and Isomerases 1% of enzyme diversity. Reductases only display 1% and Adenosine deaminases (ADA) > 1%. When considering only the total number of unique precursors, 34% of the precursors were associated with other enzymes, including phosphatases, metalloproteases or putative housekeeping enzymes, which are each represented in small amounts.

While exploring transcript diversity has already provided a good overview of putatively contained venom compound classes, a quantitative metric such as the expression level could indicate which transcripts may be more relevant in biological processes (Fig. 1B). Enzymes comprised 46% of the summed-up TPMs. OBPs (D7 proteins) accounted for 13% of the transcript abundancy while protease inhibitors accounted for 11%, and immunity-related proteins for 8%. The Antigen-5 family claimed, 4% and EGF-like proteins 2% of TPMs. Mucins remained only slightly represented with 1% of the TPMs. Multiple other protein families that may have functions in the blood feeding process were detected in the transcriptome and are referred to as “Other: salivary restricted”. However, they together account for only ca. 4% of TPMs. This group contains, for example, 23.4 kDa salivary proteins, which are known to be secreted in saliva and upregulated in blood-fed individuals and have been reported in mosquitoes^31,32^. Lastly, 13% of the TPMs represented proteins likely employed for other functions, including housekeeping and basic cellular functions and are referred to as “Other: not salivary restricted”. When focusing only on enzyme transcripts, we observed that hydrolases were most abundant and corresponded to 22% of the TPMs. These were followed by serine proteases with 21%, apyrases with 17% and peptidases with 7%. Reductases regard 6%, while isomerases and adenosine deaminases display 5% of abundancy each. Glycosidases constitute only 3% of the total enzyme expression. Various other enzyme classes, such as metalloproteases or chitinases, can be found and feature 17% of the total enzyme abundancy, while each individual annotated enzyme class within the “Other” group contains less than 3% of the TPMs.

### Proteomic integration reveals a complex venom in *Ae. albopictus*

While transcriptomic analysis reveals the entirety of transcripts expressed in the salivary glands, proteomic profiling can confirm which components are actually deployed in the venom. With this approach, the active molecular arsenal of the mosquito venom can be characterized^33^. Hence, we supplemented our salivary gland transcriptomic data with a high-resolution venom proteome for *Ae. albopictus* that used the generated transcriptome as a species- and tissue-specific database for peptide searches (Supplementary Data).

**Fig. 2:**
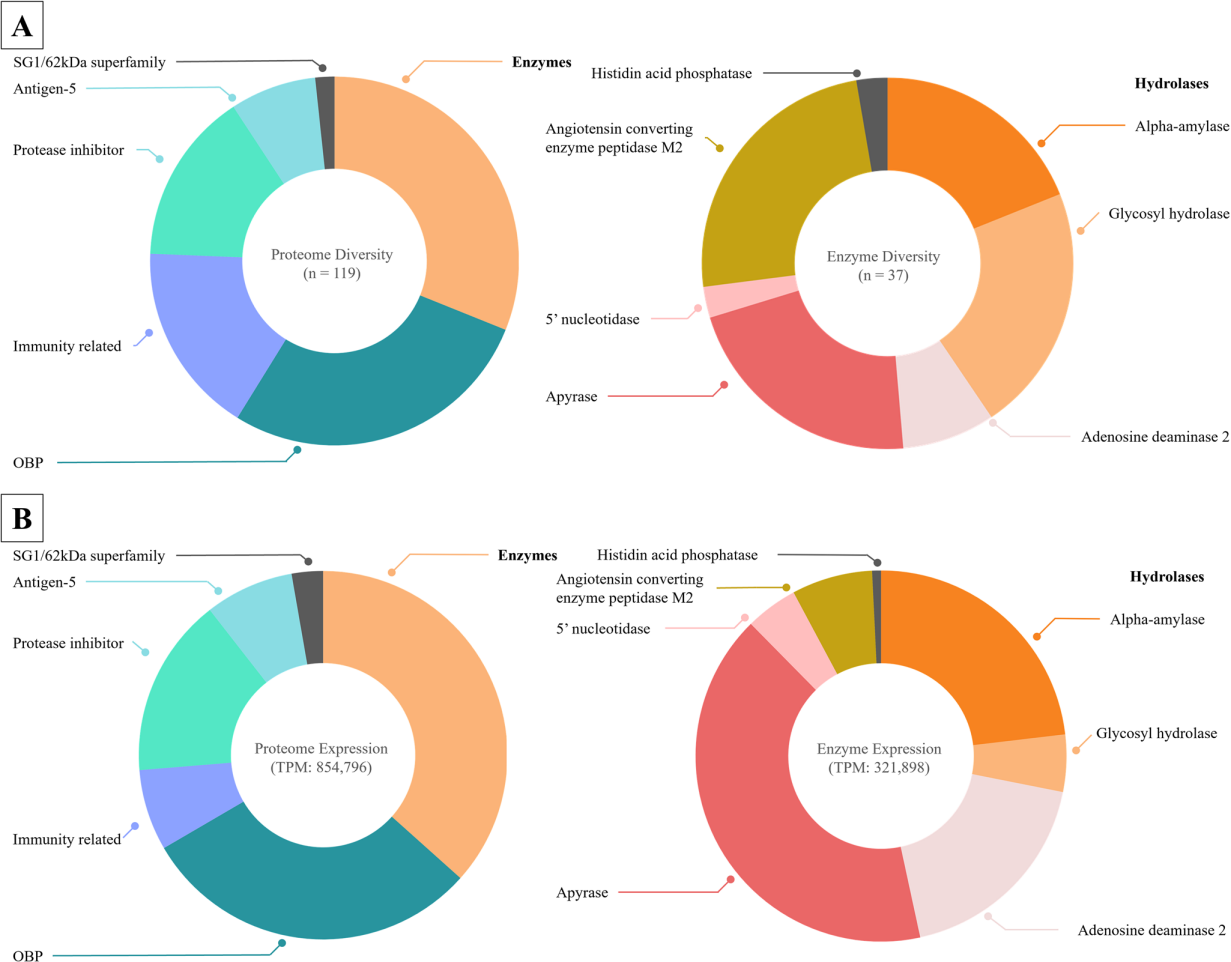
Venom proteome of *Aedes albopictus* by protein family. **A** depicts diversity. The left panel highlights the whole proteome of 119 proteins in five main classes. The right panel shows the largest group, the enzymes, with a total of 37 proteins in seven main classes. **B** The whole proteome based on expression levels from the transcriptomic data for each protein group. The left panel focuses on the whole proteome, with a total of 854,796 TPMs and the right panel displays only the enzymes, with 321,898 TPMs.

Matching the proteomic data with the transcriptome database revealed 119 precursors with proteome coverage classified to various putative protein families. A majority of the proteins identified were enzymes, which constituted 31% of all the proteins identified (Fig. 2A). OBPs, comprising 28% of all proteins, are also very prominent and consist of short and long D7 proteins. The third most diverse group are immunity-related proteins, occupying 17% of the proteome. These were annotated as members of three different families: C-type lectins, Fibrinogen-Related Domain (FReD)/ficolin domain-containing proteins, and glycoside hydrolases (lysozymes). Protease inhibitors display 15% of the proteome, consisting of serpins, cystatins and kazal-type inhibitors. Proteins of the antigen-5 family represent 8% of the proteome. Finally, 2.8% of the proteins found in the proteome could be annotated to the SG1/62 kDa superfamily. A more in-depth look at the identified enzymes revealed four main classes. First, hydrolases, which constitute 49% of all identified enzymes, are divided into three groups: glycosyl hydrolases (22%), alpha-amylases (19%) and adenosine deaminases (8%). Second, apyrases accounted for 22% of the enzymes. One potential 5’-nucleotidase was detected, corresponding to 3%. Angiotensin-converting enzymes (peptidase M2) account for 24% of all enzymes. Finally, the histidine acid phosphatase family/inositol polyphosphate phosphatase 1 was recovered with 3% of the total enzyme amount.

In addition to proteome diversity, we analyzed how prominently each protein is expressed based on TPMs (Fig. 2B) to further identify key proteins that may drive venom biology. The 119 different proteins related to a total of 854,796 TPM of our transcriptome. Enzymes represent 37% of the TPMs; OBPs (D7 proteins) represented 30%; and Protease inhibitors, 16%. Antigen-5 proteins were related to 8% of all TPMs, while immunity-related proteins correspond only to 7% of the TPMs. The remaining 3% were related to the SG1/62 kDa superfamily. When focusing only on enzymes, hydrolases are presented by a total of 46% of TPMs split into three groups: alpha-amylases (23%), adenosine deaminases (18%) and glycosyl hydrolases (5%). Apyrases represent 45% of TPMs, and 5% are classified as 5’-nucleosidases. Only 7% of TPMs are Angiotensin-converting enzymes (peptidase M2). Histidine acid phosphatase family/inositol polyphosphate phosphatase 1 are found only as 1% of the enzyme transcripts.

### Discovery of *Ae. albopictus* venom cecropins and their calculated properties

From a biodiscovery perspective, short linear peptides and immunity-related compounds are of high interest due to several aspects^34^. In addition to their roles in innate immunity, they could also play roles in modulating the host response and the microbiome and may affect the blood feeding process, vector competence and disease transmission^35–37^.

**Fig. 3:**
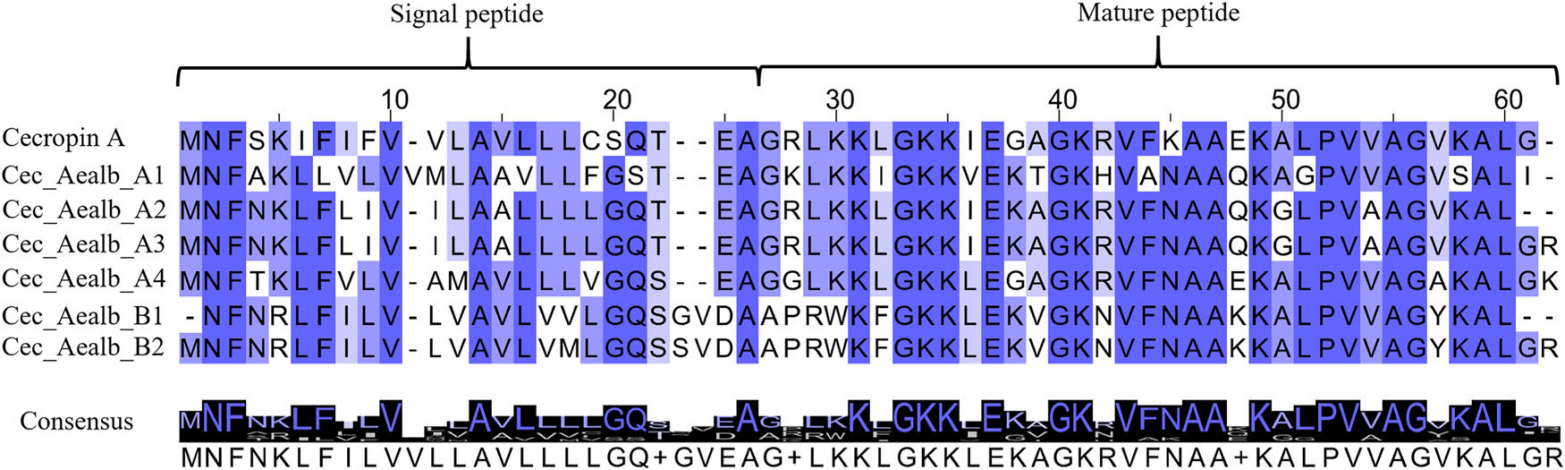
Alignment of identified cecropin precursors with cecropin A, best alignment hit cecropin from mosquitoes (UniProtKB P82290). Dark purple indicates 100%, purple 80%, and light purple 60% similarity. The noncolored amino acids indicate less than 60% similarity.

Within the transcriptomic diversity of *Ae. albopictus* salivary glands, various immunity-related compounds were identified (Figs. 2 and 3), some of which could be classified as members of known AMPs, for example, defensins or attacins. Within these our analysis indicated the presence of six peptide precursors that appear to represent members of the cecropin family. Reminiscent to other recent works that have reported cecropins from *Ae. albopictus* transcriptomes and cell lines^25,28,38^. To explore their putative functional space, we analyzed their physicochemical properties based on their primary structure to infer further details about their potential function and translational exploitability. The precursor sequences Cec_Aealb_B1, Cec_Aealb_A4 and Cec_Aealb_A3 showed more than 90% sequence similarity to previously reported mosquito cecropins from other Culicidae species (e.g., *Anopheles arabiensis* and *Aedes aegypti*), supporting our initial classification. Sequence analysis and comparative alignments revealed that the herein discovered cecropin precursors can be divided into two families. The A family (Cec_Aealb_A1, Cec_Aealb_A1, Cec_Aealb_A3) and the B family (Cec_Aealb_B1, Cec_Aealb_B2). Overall, both families exhibited high sequence similarity but higher degrees of sequence disparity at their C-terminus (Fig. 3).

For in silico analysis, we focused on putative mature peptides and their potential modifications inferred from the precursor sequence data (Table 1, Fig. 3). Different in silico tools provided insights into mature peptide properties and sequence-structure relationships. Antimicrobial Peptide Scanner vr.2 indicated that all six have a very high probability of displaying AMPs and that all could form amphipathic α-helices (Table S1). The mature peptides ranged from 28 to 36 amino acids in length, with theoretical masses ranging from 3.5 kDa to 3.9 kDa. All the *Ae. albopictus* cecropins are highly cationic according to the analysis of their primary structure: the mature peptides mCec_Aealb_A3_amide and mCec_Aealb_A3 abound with a net charge of +10. mCec_Aealb_B2_amide and mCec_Aealb_B2 had a total charge of +9, while mCec_Aealb_A1 and mCec_Aealb_A4 had a total charge of +7. The percentage of hydrophobic amino acids ranged from 42–47%, and the number of hydrophobic amino acids constructing the helices hydrophobic surface was 9 for mCec_Aealb_B2_amide and mCec_Aealb_B2, 10 only for mCec_Aealb_A4, and 12 for the others (Table 2).

**Table 1:** Names of cecropin precursors and the synthetized mature peptides

Precursor	Peptide	Sequence
Cec_Aealb_A1	mCec_Aealb_A1	GKLKKIGKKVEKTGKHVANAAQKAGPVVAGVSALI
Cec_Aealb_A2	mCec_Aealb_A3_amide	GRLKKLGKKIEKAGKRVFNAAQKGLPVAAGVKAL*
Cec_Aealb_A3	mCec_Aealb_A3	GRLKKLGKKIEKAGKRVFNAAQKGLPVAAGVKALGR
Cec_Aealb_A4	mCec_Aealb_A4	GGLKKLGKKLEGAGKRVFNAAEKALPVVAGAKALGK
Cec_Aealb_B1	mCec_Aealb_B2_amide	APRWKFGKKLEKVGKNVFNAAKKALPVVAGYKAL*
Cec_Aealb_B2	mCec_Aealb_B2	APRWKFGKKLEKVGKNVFNAAKKALPVVAGYKALGR

The sequences of synthetized peptide are given, asterisks indicate a C-terminal amidation.

**Table 2:** Physicochemical properties of the investigated cecropins

Peptide	Mass kDA	#AS	Charge	h.%	h.surf	α-helical
mCec_Aealb_A1	3.497	35	+7	43%	12	**+**
mCec_Aealb_A3_amide	3.546	28	+10	44%	12	**+**
mCec_Aealb_A3	3.759	36	+10	42%	12	**+**
mCec_Aealb_A4	3.576	36	+7	44%	10	**+**
mCec_Aealb_B2_amide	3.728	34	+9	47%	9	**+**
mCec_Aealb_B2	3.941	36	+9	44%	9	**+**

The table displays the name, with the peptides mass, number of amino acid residues (#AS), charge, hydrophobicity (h%), number of hydrophobic AS condensing on one side of the helix (h.surf), and their predicted ability to form amphipathic α-helices.

### Structural dynamics of cecropins

**Fig. 4:**
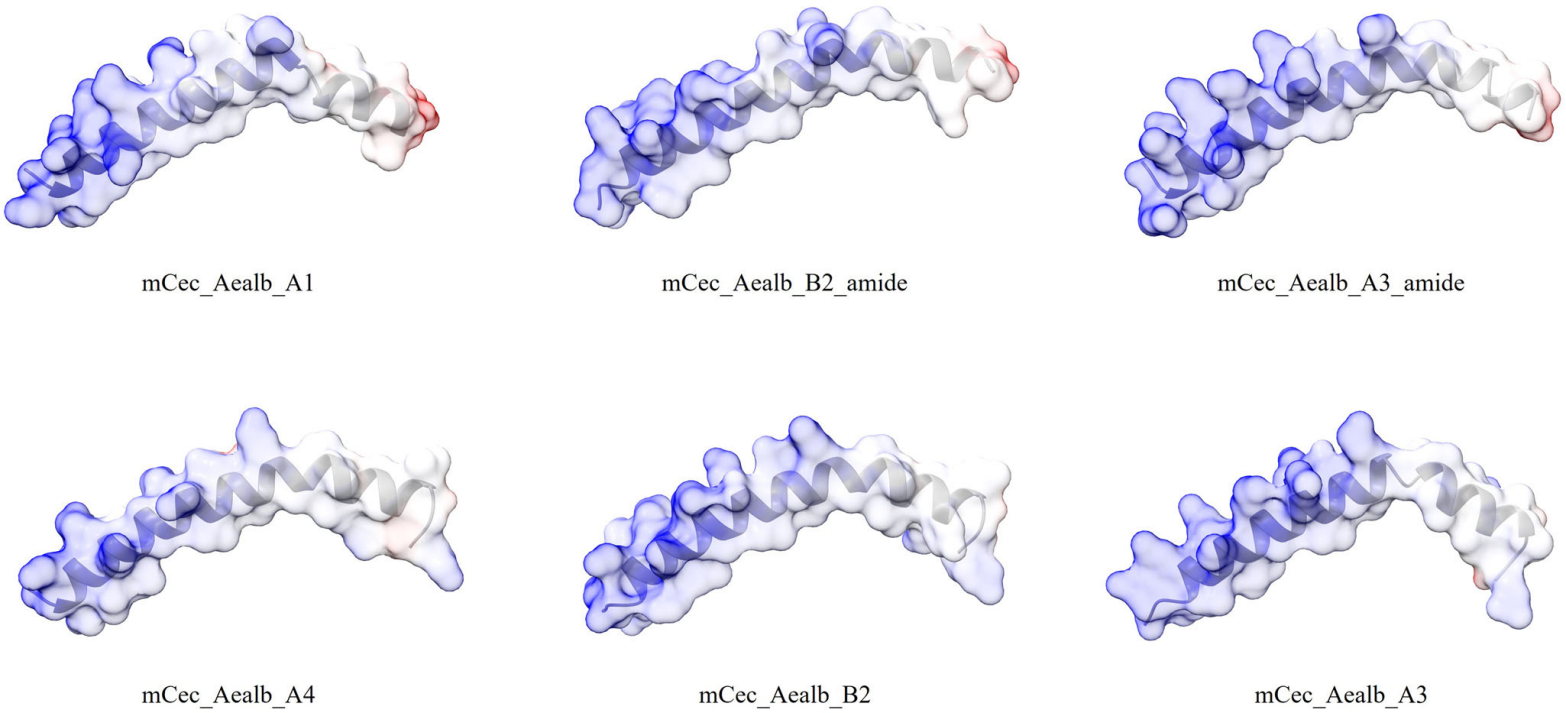
3D structure of cecropins as predicted by Alphafold3. The α-helical organization of the peptides is presented in gray. Additionally, the peptide surface is displayed with red coloration for negative charge, white for neutral charge and blue indicating a positive charge. Models were created with UCSF ChimeraX 1.10.

Our structural analysis further supports that the cecropins fold into α-helices. All showed a hinged C-terminal end at amino acid positions 23 to 26. In most peptides, these regions contain proline and glycine. The distribution of hydrophobic amino acids occurs mainly on one side of the helices (Fig. S1). Interestingly, all the peptides developed a positively charged surface towards the N-terminus. A negative charge can be found at the C-terminal region (Fig. 4).

### *Ae. albopictus* venom cecropins have anti-*E. coli* activity

The structural and physicochemical properties as well as the literature on insect-derived cecropins prompted us to test the biological activities of the homologs identified herein, starting with their antimicrobial traits. Therefore, putative mature peptides of the precursors were synthesized with their likely modifications inferred from the sequence data (Table 1). Two cecropins precursors occur as the same putative peptide with and without a C-terminal glycine, which serves as an amidation signal. This indicates that the resulting peptide may be the truncated amidated version, respectively. Hence, for the peptides precursors, for which we detected full-length and truncated versions, we synthesized the unprocessed and amidated mature peptides (precursors Cec_Aealb_A2/A3 for mature peptides mCec_Aealb_A3_amide and mCec_Aealb_A3; as well as, Cec_Aealb_B1/B2 for mCec_Aealb_A2_amide and mCec_Aealb_A2) (Tables 1 and 2). For the other peptides, we synthesized the putative peptides as indicated by theor sequence.

A first pre-screening of all six synthesized mature structures of cecropins against *E. coli* DSMZ 102053 showed strong inhibitions of its growth over the course of 48 h at a concentration of 200 µM (Fig. S2, Table S2). This led us to further investigate the antimicrobial effects of these compounds against a larger panel of microorganisms and to calculate their MIC. The tested bacterial strains included *E. coli* ATCC25922, *M. smegmatis* ATCC607, the methicillin-resistant *S. aureus* strain ATCC33592 and the yeast *C. albicans* FH2173. None of the tested peptides had MICs lower than 50 µM against *S. aureus* or *C. albicans*, hence indicating little to no activity. For *M. smegmatis*, only mCec_Aealb_B2_amide seems to be active, with a MIC ranging from 25 to 12.5 µM. In contrast, strong activities were found against *E. coli*, with the exception of mCec_Aealb_A1 with a MIC of 50 µM. mCec_Aealb_A4 exhibited activity at concentrations ranging from 6.25 to 3.125 µM. The three cecropins mCec_Aealb_B2_amide, mCec_Aealb_B2 and mCec_Aealb_A3 had very low MICs of 0.4 µM, while mCec_Aealb_A3_amide had inhibitory effects at 0.2 µM (Table 3).

**Table 3:** MIC of cecropins

Peptide	*E. coli*	*E. coli* (+LPS)	*M. smegmatis*	*S. aureus* (MRSA)	*C. albicans*
mCec_Aealb_A1	50 µM	nd	>50 µM	>50 µM	>50 µM
mCec_Aealb_A3_amide	0.2 µM	3.125 µM	>50 µM	>50 µM	50 µM
mCec_Aealb_A3	0.4 µM	6.25 µM	>50 µM	>50 µM	>50 µM
mCec_Aealb_A4	6.25–3.125 µM	6.25 µM	>50 µM	>50 µM	50 µM
mCec_Aealb_B2_amide	0.4 µM	6.25 µM	25-12.5 µM	>50 µM	50 µM
mCec_Aealb_B2	0.4 µM	12.5 µM	>50 µM	>50 µM	>50 µM

The investigated cecropins were screened against *Escherichia coli* ATCC 25922, *Mycobacterium smegmatis* ATCC 607, *Staphylococcus aureus* (MRSA) ATCC 33592, and *Candida albicans* FH2173. The lowest concentration at which the strains inhibited growth was provided for each tested strain.

These strong effects against *E. coli* led us to further investigate the activity of selected peptides in presence of LPS. mCec_Aealb_A1 was excluded from this test because it lacked antibacterial effects. When tested in the presence of LPS, the activity of all tested cecropins decreased 15 to 30-fold. mCec_Aealb_A3_amide exhibited activity at 3.125 µM in the presence of LPS, while mCec_Aealb_B2_amide, mCec_Aealb_A4 and mCec_Aealb_A3 exhibited an inhibition range reduced to 6.25 µM, and that of mCec_Aealb_B2 was reduced to 12.5 µM.

### *Ae. albopictus* venom cecropins show weak effects on mammalian cells

Given the antimicrobial properties observed, we next investigated whether the peptides exert cytotoxic or hemolytic activity. This will provide further insights into the biology of the peptides and highlight limits of potential therapeutic applicability.

**Fig. 5:**
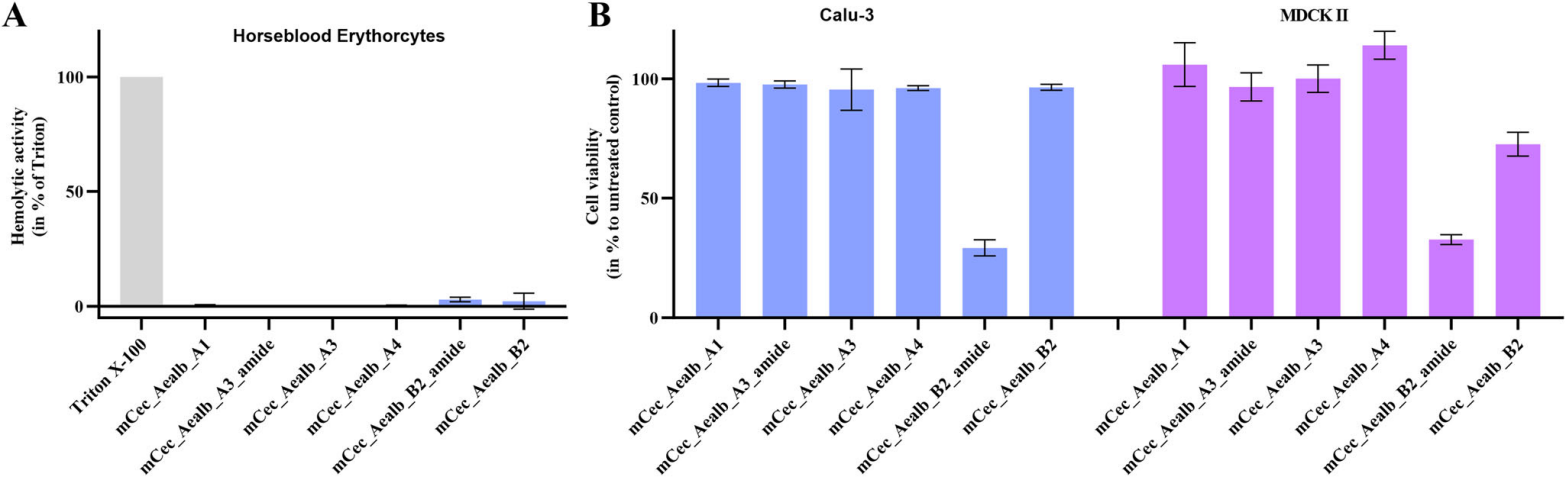
Cytotoxic effect of the cecropins on vertebrate cells. **A** Hemolytic activity of cecropins against horse blood erythrocytes at a concentration of 200 µM. The bar chart displays hemolytic activity for each cecropin after 1 h of incubation with erythrocytes. The data were normalized to the hemolytic activity of Triton X-100 as 100% of activity and to that of the solvent DMSO in 1:5 dilution as 0%. **B** The left bar chart represents Madin-Darby canine kidney cells (MDCKII) (blue), and the right bar chart represents human-derived airway epithelial cells (Calu-3) (purple). The bars show cell viability after 48 h of treatment with the cecropins. All experiments were carried out in triplicate, and the data is displayed as means with standard deviation. Charts were created using GraphPad Prism 10.0.2.

First, we assessed the activity of the peptides against horse blood erythrocytes. The tested cecropins had no hemolytic effect on the tested blood cells at a concentration of 200 µM. We further tested the effects of cecropins on two vertebrate cell lines, MDCKII and Calu-3. In both cell lines, the peptides mCec_Aealb_A1, mCec_Aealb_A3_amide, mCec_Aealb_A4 and mCec_Aealb_A3 showed no noteworthy decline in cell viability. In MDCKII cells, mCec_Aealb_B2 displayed a reduction of cell viability of ~30% compared to the untreated control. mCec_Aealb_B2_amide reduced the viability of MDCKII and Calu-3 cells by ~70% (Fig. 5).

## Discussion

Given the critical role of mosquitoes as vectors, a precise understanding of the biochemical complexity of their venom is essential. To achieve this, a proteotranscriptomic-guided modern venomics experiment was employed to disentangle the molecular arsenal present in the venom of the Asian tiger mosquito (*Ae. albopictus*). We report another perspective to mitigate potential biases inherent in conventional sialotranscriptomic approaches. This strategy allowed us to explore the components of mosquito venom, which may play key roles in blood feeding, pathogen transmission, and host manipulation while also serving as potential targets for vector control and as sources of novel therapeutic agents. Our proteo-transcriptomic investigation of the *Ae. albopictus* venom system was guided by the sialome nomenclature established by Ribeiro et al. (2010) and VectorBase^39^ paired with the venom databases VenomZone (available online: https://venomzone.expasy.org/) and Tox-Prot^40^. Overall, our findings align with previous pioneering works of Ribeiro, Arca and colleagues, which paved the foundations of sialomic studies in hematophagous dipterans^15,32^. Considering the totality of the so far existing data, Mosquitoes appears to abound with a chemically and functionally surprisingly complex venom, with several shared protein families that may be of importance^32,41^. This includes families, such as D7, antigen-5, apyrase, and ADA, which are repeatedly found in various Mosquito venoms and deserve additional scientific attention^32,42–44^. However, our study refines the current understanding of the molecular arsenal of the *Ae. albopictus* venom system and is followed by a discussion of the particularities and potential roles of the most abundantly represented components of our analysis.

Proteomic analysis revealed the diversity and functional space of *Aedes albopictus* venom enzymes. Enzymes constitute a major portion of mosquito venom. The largest enzymatic group comprises hydrolases, a diverse class known to participate in processes such as feeding, digestion, and reproduction in arthropods^45^. Alpha-amylases and glycosyl hydrolases were found in high abundance and were present across all blood-feeding Nematocera. They may display the evolutionary link to the conserved ancestral trait of sugar feeding and the independent evolution of hematophagy between members of Culicomorpha and Psychodidae^42^. Another prominent enzyme group comprises ADA, which is proposed to facilitate blood feeding via anti-hemostatic and anti-inflammatory activities and to act as immunomodulatory agents^42,46^. Notably, ADA has also been linked to enhanced dengue virus replication in *Ae. albopictus*^47^. Apyrases and 5′-nucleotidases were identified which hydrolyze ATP and ADP, which are key components of the blood coagulation cascade and thereby may facilitate blood uptake by perturbing blood clotting^20,42^. We also detected a high diversity of ACE homologs, which are likewise considered as blood-feeding facilitators based on their role in vertebrate blood pressure regulation^48–50^, raising the intriguing possibility that mosquitoes might exploit this function to maintain blood flow during feeding, although this hypothesis has yet to be experimentally validated. Another identified group of feeding-related enzymes are phosphatases, primarily histidine acid phosphatases^51^. To our knowledge, this is the first report of phosphatases in mosquito venom. Their role may involve blood feeding by hydrolyzing dinucleotides, thereby suppressing platelet-derived inflammatory mediators, which remains to be further investigated^52,53^. In sandflies, they have been shown to inhibit host coagulation proteins, and similarly, in cattle ticks, elevated phosphatase activity has been observed during blood feeding^54,55^. Overall, our analysis revealed *Ae. albopictus* venom as a rich source of enzymes, similar to the venoms of other neglected arthropods as suggested by recent studies^56–59^. Considering their biological function, many of those are associated with blood feeding and thus serve a primarily trophic function. Additionally, multiple of the enzyme families proposed herein could be targeted for vector control to inhibit blood feeding efficiency to disturb other physiological processes within mosquitoes or as biomarkers^60,61^.

In addition to the array of enzymatic components, we detected several components from various non-enzymatic protein families across our generated *Ae. albopictus* venom proteome. Henceforth, we discuss these components in light of their known biological activities and their potential role in the venom system. One major protein group is OBPs, which are primarily represented by D7 salivary proteins. These are well characterized in mosquito venom and are present in other blood-feeding Diptera^10^. D7 proteins are grouped into long and short forms, containing one or two OBP-like domains with six α-helices and three disulfide bonds each. OBPs can be found in the antennae, where they may facilitate olfactory functions^10^. The function of the sialomic analogs (D7 proteins) function was first described by James et al. (1991) in *Ae. aegypti* salivary glands as supporting the feeding capabilities of female mosquitoes^43^. Since then, they have been reported and described regularly within mosquitoes^62^. The first specific D7 protein was described by Valenzuela et al. (2003) in Anopheles stephensi and was suggested to play roles in both sugar and blood feeding by inhibiting factor XII^63^. Isawa et al. (2002) characterized Hamadarin, a D7 protein with anti-hemostatic and anti-inflammatory properties, as a promising candidate for anti-inflammatory and analgesic drug development^62,64–66^. D7 proteins likely contribute to host detection, but they also exert direct effects during blood feeding. The C-terminal domains of these proteins can bind biogenic amines and host mediators such as serotonin, histamine, and norepinephrine, which are molecules that induce vasoconstriction, platelet aggregation, itching, and pain. Inhibiting these mediators promotes painless and efficient blood feeding^64^. The N-terminal regions may be involved in suppressing vasoconstriction or interfering with the coagulation cascade^44^. D7 proteins affect pathogen survival and transmission. They bind dengue virions, inhibiting viral replication and infection both in vivo and in vitro^67,68^. This dual role presents them as a two-edged sword: while they may protect mosquitoes from infection, they can simultaneously suppress host immune responses, potentially increasing disease transmission risk. These contrasting effects make D7 proteins compelling targets for further investigation in medical and vector control contexts. Another well-known mosquito venom component, mucin, has surprisingly been detected in only small amounts within the transcriptome, even though it is usually recalled as common in the sialotranscriptomes of mosquitoes and other hematophagous insects^63,69,70^. These proteins are proposed to act as lubricants in the food canal^32,70^. Their absence from the proteome and low expression in the transcriptome may suggest rapid degradation, low translational activity or a less important role for the venom, as previously expected.

Several immunity-related protein families were identified in the venom proteome. C-type lectins (CTLs) may have paradoxically two converse effects: (1) protection against microbial infection during feeding as components of the mosquito’s innate immune system^71^ and (2) enhancement of arbovirus infection and replication, as shown in *Ae. aegypti*, by binding to viral envelope proteins^72^. Other immunity-related peptides include FReD proteins and ficolins described from the salivary glands and hemolymph of several arthropods. These molecules are upregulated following exposure to bacteria or arboviruses and may function in pathogen recognition^73^. Additionally, cecropins are known antimicrobial peptides involved in mosquito immune responses and possibly in venom function. Another group are protease inhibitors, which show high diversity in the mosquito venom. Proteases such as thrombin and trypsin are essential for vertebrate hemostasis and immune responses^74^, and their inhibition is a key element to support blood feeding. Common protease inhibitors in our dataset included Kazal-type inhibitors, serpins, cathepsins and cystatins. Among these, cathepsins stand out as multifunctional protease inhibitors that may facilitate blood digestion and amino acid release for egg development^75,76^. Furthermore, in *An. gambiae*, cathepsins have been shown to exert negative effects on *Plasmodium* parasites, and in *Aedes* species, correlations with dengue virus titers have been reported^77,78^. Another family found in the proteome is the CAP superfamily, particularly antigen-5 peptides. Although these peptides are widely distributed among blood-feeding insect venoms, no functional characterization of CAP from mosquito venom has been carried out thus far. However, in hematophagous triatomine bugs, CAP peptides are thought to inhibit collagen-induced platelet aggregation and ATP release^44,79^. A similar role could be speculated for *Ae. albopictus* derived CAPs, but this hypothesis requires more extensive research and confirmation. Additionally, a minor protein group identified in the proteome was the SG1/62 kDa superfamily, which included the secreted salivary protein SGS1. Besides being a protein group identified only as a minor fraction, we were able to confirm its presence at the protein level. The literature describes the 62 kDa family as exclusive to *Aedes* species^32^, and our data validates them on protein level in the *Ae. albopictus* venom system. They are of particular interest due to their proposed role in pathogen transmission^80^. Strikingly, we did not find members of the 34 kDa protein family, which are known to be of pivotal importance during the blood feeding process of mosquitoes^23,32^. This may be because we analyzed 5-day-old mosquitoes, while previous studies reporting these proteins relied on older specimens^23^. Taken together, these findings suggest that non-enzymatic proteins are rich sources of biomolecules that play roles in pathogen transmission or blood feeding. The non-enzymatic may further serve as biomarkers for mosquito exposure. For instance, the 62 kDa superfamily could be a biomarker candidate because its members show unique sequence patterns in metazoans and they are quite restricted to the *Aedes* venom system^32^. A thorough understanding of mosquito venom and its non-enzymatic elements may thus form the basis of future pharmacological innovations or devices^15,32^.

We focused primarily on six components that were classified into the AMP family of cecropins, one of the largest and most studied families of insect AMPs^81^. Typically, by adopting an α-helical structure, these compounds are known for their potent activity against both gram-negative and gram-positive bacteria while exhibiting low toxicity and even anti-inflammatory effects in mammalian systems^37,82,83^. In addition to antibacterial activity, cecropins have shown efficacy against fungi, protozoa, and viruses^84–87^. Even antimalarial effects of cecropins were reported in *Anopheles* mosquitoes^88^, and their role in microbial clearance during pupation has been highlighted^24^. In addition, cecropins possess anti-inflammatory properties which they exert by binding to bacterial membranes. Various insect-derived cecropins have been investigated as potential candidates for therapeutic applications^81,89^.

Structural predictions and bioinformatic analysis of the cecropins identified in this study revealed the presence of α-helical conformations, a high net positive charge, and a distinctly amphipathic nature with hydrophobic amino acids predominantly localized on one side of the helix. These features are consistent with typical membrane-active linear cytolytic AMPs and suggest that these peptides may disrupt microbial membranes through pore formation or lipid bilayer destabilization^90,91^. A particularly interesting structural element is the helix breaking proline after the N-terminal amphipathic helix region, leading to a hinged hydrophobic and negatively charged C-terminal domain. This organization is common for cecropins and might be crucial for their interaction with bacterial membranes^81,92–94^. This architecture points to a well-described mechanism of action referred to as “type II channel formation”. According to this model, the positively charged C-terminal region anchors the peptide to the negatively charged outer membrane surface, primarily via interactions with LPS, while the hydrophobic hinge region inserts into the membrane. As multiple peptides aggregate, they form stable transmembrane pores that compromise membrane integrity and ultimately lead to cell lysis^90,91^. The structural predictions suggest that the cecropins in *Ae. albopictus* venom likely conform to this same type II channel mechanism. Their predicted amphipathic and electrostatic profiles support membrane interactions, and the presence of a defined hinge region further points toward potential pore formation, as documented for other insect-derived cecropins (Fig. 4)^94^. Based on these structural cues, we investigated the activity of these compounds via functional screenings against several microbial strains. The antimicrobial screenings of *Ae. albopictus* cecropins revealed that these compounds strongly inhibited the gram-negative bacterium *E*. *coli*, with MICs as low as 0.2 µM. This confirmed our expectations, as their bacterial membranes structurally differ from those of gram-positive bacteria, where no activity was detected. Interestingly, mCec_Aealb_A1 had weaker antibacterial effects than the other cecropins while still yielding high charge and hydrophobicity. This difference may be explained by the unique sequence features of this peptide compared to those of its relatives. This includes the lysine at position 2 or the histidine at position 16, among others. In venom components and AMPs, single amino acid exchanges can exert tremendous functional effects on peptides^95,96^. Therefore, carrying out alanine scanning on the various cecropin A family members would be an interesting avenue for future research to infer the functional and structural importance of specific residues along the peptides primary structures.

To gather further insights into the mode of action of the cecropins, we explored their interaction with LPS, a major component of the outer membrane of gram-negative bacteria. Membrane-disrupting AMPs generally bind to LPS in bacterial membranes as an initial step in their disassembling mode of action^97^. The screening of antimicrobial growth inhibition in the presence of free LPS highlighted that the antimicrobial effect of the tested cecropins against *E. coli* was reduced by a factor of 15 to 30. This finding suggested that the cecropins bind to free LPS, reducing the amount of cecropin available to bind bacterial membranes. These results support the hypothesis drawn from structural analysis that these peptides act via a membrane targeting mechanism. To further characterize the biological function of the cecropins and to evaluate the pharmaceutical potential of these peptides, we explored their activity on various mammalian cells. Specifically, we tested two mammalian epithelial cell lines and erythrocytes. In particular, the latter are a common first-line system estimate for potential side effects caused by AMPs. In our hemolytic activity screening, none of the tested cecropins displayed hemolytic effects. Cytotoxicity screening revealed that only mCec_Aealb_B2_amide exhibited cytotoxic effects on epithelial cells. mCec_Aealb_B2 exhibited toxic effects on Calu-3 cells. The cytotoxic effects of the Cec_Aealb_B-family members may be exerted by their distinct N-terminal organization, which makes them unique from the A-family members. The other compounds showed no noteworthy cytotoxicity. Given the potent anti-*E. coli* activity of these cecropins and the absence of cytotoxic or hemolytic effects, we identified these peptides as promising for further exploration of their pharmaceutical potential. Taken together, the tested cecropins appear to function as membrane-active, potentially pore-forming AMPs with strong activity against gram-negative bacteria such as *E. coli* but not against other microbes or mammalian cells. Since some cecropins have shown to act synergistically, future studies should explore combinations of cecropins to provide a more conclusive picture of their function and bacterial targeting ability.

Nonetheless, our study is affected by some limitations that need to be taken into account when interpreting our findings. First, the taxonomic coverage of studied Mosquito venom systems, into which our work is embedded, represents an intrinsic limitation towards interpretation of our findings. Hematophagous dipterans, as a whole, but particularly mosquitoes, are a diverse group of insects that form multiple evolutionary lineages and ecotypes^98^. Many are important vectors of pathogens, and their venom systems are key elements in the transmission process. That said, only a small fraction of the ca. 3500 species within Culicidae (Mosquitoes and their allies) has been studied in this regard^99^. In our work, we shed light on the biochemical profile of the *Ae. albopictus* venom system and found similarities to the few other previously analyzed venoms, which revealed, e.g., Hydrolases, Apyrases, and OBP(D7-family) as commonly expressed major components across various taxa^32^. However, before any general conclusions about the relevance of specific components in mosquito venoms can be drawn, the number of Culicidae species studied from a venomics perspective needs to increase. Else, overinterpretation of the available small fraction could give rise to a form of taxonomic bias, as commonly discussed in arachnological toxinology^100^. A second limitation lies in the sampling process. We analyzed non-blood-fed specimen aged 5 days, while some studies partially analyzed specimens at later stages, others at earlier stages, and some used blood fed animals, which limits the comparability between studies^23,101,102^. In many venomous lineages, ontogenetic venom variation (variation in venom composition over time and life history stages) is well-established^103,104^, but whether this process manifests in Mosquitoes is currently unclear, and understanding this would be an important future task. Conceptually, it is reasonable to assume that if venom variation occurs in Mosquitoes, divergent venom profiles will be created within only a few days considering the short life span of most adults and their prioritized sugar feeding in the first days of emergence^105^. This could explain some of the discrepancies observed. Another potential impediment is the milking process. Venom collection from small invertebrates is extremely challenging and often fails completely^17,30^, rendering transcriptomics the most reliable source of information for many arthropod venom systems^30^. Here, we successfully milked crude venom by using osmotic pressure from mineral oil and subsequent isolation of the hydrophilic venom components. Nevertheless, at the time of writing, it is unclear whether our isolation procedure is absolute and equally effective for every component, and some may be retained in the oil fraction and hence remain absent from the proteomics experiment. In light of that, our proteomic analysis should be understood as a conservative perspective. The full *Ae. albopictus* venomic arsenal may be even greater than that reported herein. A final caveat revolves around the identification of cecropins, which we have identified at the mRNA level and characterized in more detail in light of the families well-known antimicrobial potential. It is important to note that we have not yet verified their existence on protein level. This stems, on the one hand, from their low abundance and diversity, which renders them difficult to detect. Second, we applied a bottom-up approach in which polypeptides are digested into smaller fractions and, in case of short AMPs, often leads to fragments that are poorly captured by mass spectrometric devices. This obstacle is often encountered in venom biodiscovery from small arthropods and often leads to the compound-inference from transcriptomic data and other sequence-based sources^30,106,107^. It would be important to employ one of the rapidly developing mass spectrometry technologies of Modern Venomics or to chemically isolate the peptides to verify their existence and their modifications on protein level^108–110^.

In conclusion, we can state that mosquitoes, especially *Ae. albopictus*, are vector insects that rely on venom to obtain a blood meal, which also facilitates the transmission of pathogens. Their venoms lie at the functional interface between host, pathogen and vector and hence plays a key regulatory role. Furthermore, considering their coagulo-modulatory and immune-related roles, mosquito venoms represent a rich source for novel biomedical leads. Nevertheless, comparatively little is known about their venom profile. Here, we fill this important research gap by carrying out an in-depth modern venomics profiling of *Ae. albopictus* venom. Our analysis revealed a chemically complex venom composed of at least 119 proteins from six protein families, that were verified at the protein level. Based on the functional annotation of the venom compounds, *Ae. albopictus* venom was found to be rich in enzymes (1018 transcripts, 37 components), but non-enzymatic families were also abundant (1356 transcripts, 82 components). With respect to compounds directly affecting blood feeding, the identified putative hydrolases, in particular, have homologs known to target the cardiovascular system of vertebrates (e.g., ACE and D7-salivary proteins). Likewise, the enzymatic families might interfere with the coagulation cascade (e.g., Apyrases, 5′-nucleotidases, phosphatases and peptidases). Our analysis revealed a plethora of biomacromolecules in *Ae. albopictus* venom that likely facilitate blood feeding and therefore add to our understanding of the biochemical basis of mosquito feeding and, consequently, their vector transmission process. We further identified a range of components with potential immune function (C-type lectins, FReD proteins and ficolins), including novel cecropin peptides. Structural in silico analysis revealed these peptides to be linear cytolytic peptides. In vitro screenings for bioactivity highlighted these peptides as potent AMPs that target gram-negative bacteria but no other pathogens nor mammalian cells. This work demonstrates the power of modern venomics, which enables the discovery of bioactive molecules that may be underrepresented or undetected by classical approaches of sialome research. This study thereby provides novel insights into venom complexity and expands the repertoire of candidates for therapeutical applications and potential targets for vector control. Future works exploring synergistic combinations and bioengineering strategies may further enhance the efficacy and application potential of these bioresources.

## Methods

### Mosquito rearing

**Fig. 6:**
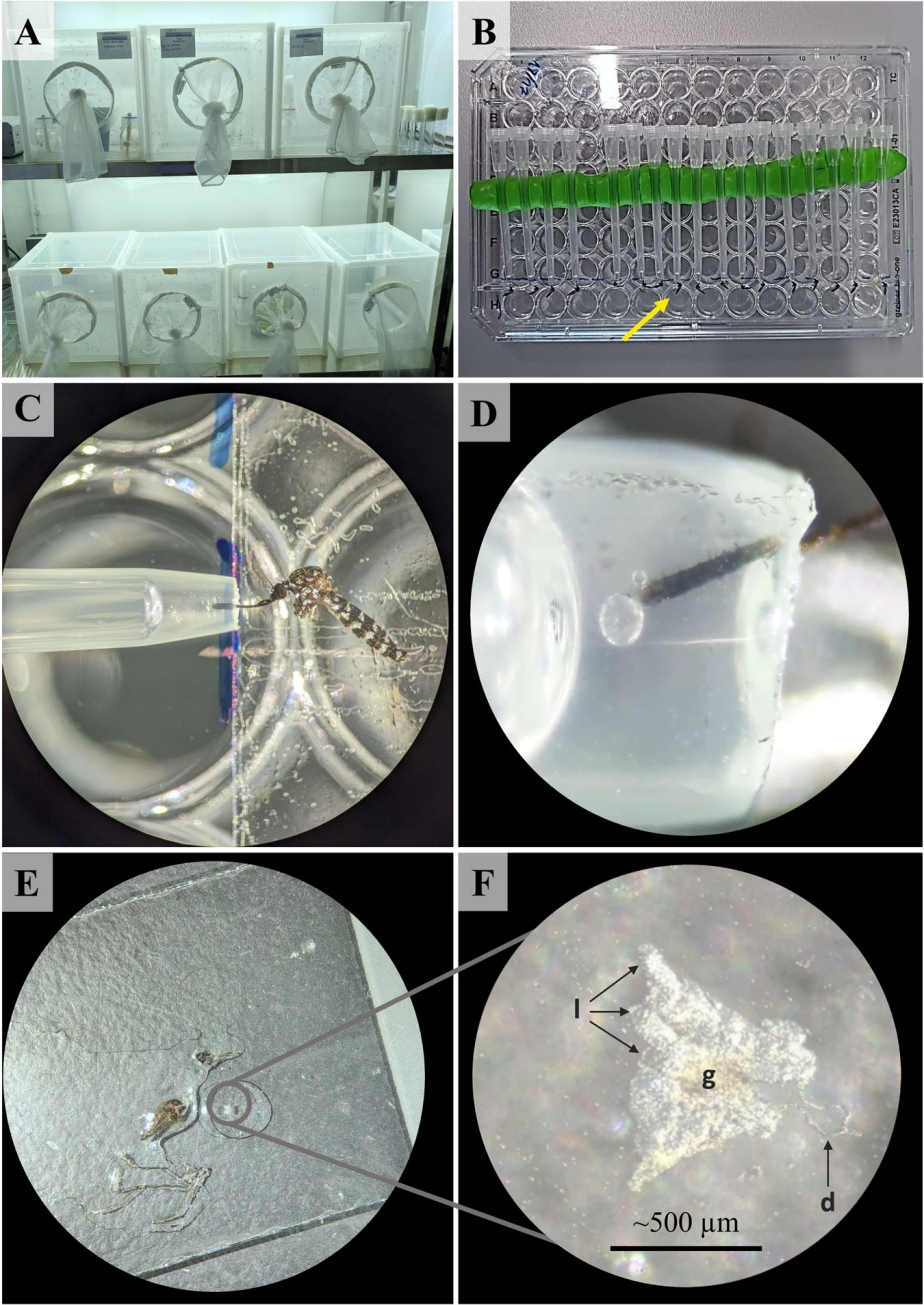
Mosquito rearing, venom collection and gland dissection. **A** Mosquitoes in the rearing cages prior to processing. **B** Immobilized mosquitoes with their mouthparts inserted into a pipet tip containing mineral oil to induce salivation and collect saliva, yellow arrow highlights the position of mosquitoes, while (**C**) is zoomed in on a single mosquito. **D** A magnified view on the pipet tip with the mosquito saliva visible in the oil. **E** A mosquito after successful dissection process. In (**F**), a magnified view of a salivary gland pair (g) connected via the salivary ducts (d) is displayed. Organization of a gland pair into three lobes is recognizable (l). Photographs are original and created by Ludwig Dersch.

*Ae. albopictus* mosquitoes (Rimini strain, kindly provided as eggs by Dr. Hanano Yamada) were reared at 28 ± 3 °C and 80 ± 10% relative humidity with a 12 h photoperiod. The animals were fed on 8% D(-)-fructose (Carl Roth) ad libitum. For rearing, the females were provided weekly with defibrinated sheep blood (Thermo Fisher Scientific) for 1 h using the Hemotek system (Hemotek Ltd) set at 37 °C. After blood meal, an oviposition site was prepared from a black plastic pot with humid filter paper and put into the rearing cage. The egg-containing filter papers were carefully dried over 10 to 14 days. Eggs were hatched in jars containing deoxygenated water. After hatching, the larvae were transferred to plastic trays containing two liters of tap water supplemented with two Pleco Tablets (Tetra) as larval feed. Over the next 5 days, new feed was provided daily. Pupae were collected in a jar and placed in a rearing cage for adult emergence (Fig. 6A).

### Salivary gland dissection

Dissections were performed on non-blood-fed 5-day-old female mosquitoes. Prior to dissections, the sugar solutions were removed from the rearing cages and animals were starved for 3 h. Individual female mosquitoes were collected from the cages and kept separated in Ø 29 × 95 mm vials with Ø 30 × 30 mm foam stoppers (Nerbe Plus). Animals were immobilized by CO_2_ and passed through ethanol to disinfect the animal surface and neutralize charges that may complicate the dissection process and then placed on glass slides with 50 µL droplets of PBS. All dissection steps were performed in PBS and under a Stemi 508 Stereomicroscope (Zeiss) at 40× magnification. For the dissection process, wings and legs were removed with extra fine straight forceps No. 5 (neoLab), then the head was carefully pulled off the thorax. Afterwards, gland pairs were precisely removed and washed by passing them through PBS (Fig. 6E, F). Gland pairs were stored and pooled in 500 µL RNA-later (Thermo Fisher Scientific) at −20 °C until further processing. In total, 60 salivary gland pairs were dissected and used for transcriptomics.

### Venom collection

For venom collection, non-blood-fed 5-day-old female mosquitoes were used. Therefore, groups of 5 animals were collected from rearing cages, kept in Ø 29 × 95 mm vials with Ø 30 × 30 mm foam stoppers (Nerbe Plus) and starved for 3 h. Before venom collection, a plate with a stripe of common double-sided adhesive tape for mosquito immobilization and modeling clay for pipet tip stabilization was prepared (Fig. 6B). Mosquitoes were cold-immobilized by placing the vials on ice for 3 min. Wings and legs were removed by forceps and the body of the mosquito was affixed on the double-sided adhesive tape. The mouthparts were carefully inserted in pipet tips filled with 2 µL of mineral oil (Carl Roth). Plates prepared with mosquitoes were placed in a climate chamber at 28 ± 3 °C and 80 ± 10% relative humidity for up to 1 h to further support passive salivation. Afterwards, droplets of salivary secretion were visible within the oil in the pipet tips (Fig. 6C, D). Pipet tips were carefully removed and all containing liquid was acquired by centrifuging for 2 min at 2000 RCF into an Eppendorf tube. For centrifugation tubes containing 20 µL of ddH_2_O and a custom-made pipet tip spin-down adapter were used. Finally, samples from 250 mosquitoes were pooled in a single tube. The tube was centrifuged at 4 °C and 20,000 RCF for 10 min to separate the venom from the oil. The aqueous phase containing water and venom was recovered and lyophilized. The sample was then stored at −80 °C until further proteomic processing.

### Proteotranscriptomics

For a detailed venomic assessment of *Ae. albobictus*, we utilized our in-house modern venomics platform established previously^111,112^. Total RNA extraction, library preparation, and sequencing was outsourced to Macrogen. SMART-Seq v4 Ultra Low Input RNA Kit for Sequencing and TruSeq RNA Library Preparation v2 Kit was used for library preparation and sequencing. The kit includes SMART (Switching Mechanism at 5′ end of RNA Template) technology to select mRNA. Sequencing was performed on an Illumina platform to produce 151 bp paired-end reads. The raw sequencing data are available under project PRJEB96266 at the European Nucleotide Archive (ENA, https://www.ebi.ac.uk/ena/). Crude venom was analyzed by bottom-up mass spectrometry. Our proteo-transcriptomic investigation of the *Ae. albopictus* venom system was partly guided by the sialome nomenclature established by Ribeiro et al. (2010) and VectorBase^39^ paired with venom databases VenomZone (available online: https://venomzone.expasy.org/) and Tox-Prot^40^.

#### Transcriptomics

Sequencing data were quality controlled with FastQC v0.11.9^113^. Sequencing adapters were removed and reads quality trimmed with cutadapt v4.2^114^ with a quality cutoff of 28 and a minimum length of 30 bp. We used a multiple assembler strategy including Trinity v2.13.2^115^ with a minimum contig size of 30 bp and maximum read normalization of 50, rnaSPAdes v3.15.5^116^ with and without reads corrected with Rcorrector v1.0.5^117^. Both rnaSPAdes assemblies were built with k-mer sizes of 21, 33 and 55. All three resulting assemblies were concatenated for further analysis as one assembly. We mapped all trimmed reads with HISAT2 v2.2.1^118^ against the assembly for quality control and used StringTie v2.2.1^119^ to calculate the transcripts per million (TPM) value for each contig. We also used samtools v1.16.1^120^ for data transformation. TransDecoder v5.5.0 (RRID:SCR_017647) was performed to extract and identify open reading frames (ORFs) with a minimum length of 10 amino acids. All ORFs without a predicted stop codon were removed. Resulting ORFs with identical length and amino acid sequence were combined with fastanrdb from the Exonerate suite v2.4.0^121^ and handled as single ORFs with the corresponding TPMs summarized and kept for the united ORF. We screened all remaining ORFs with InterProScanv5.61-93.0^122^ including all databases available. ORFs without an associated InterPro entry nor a predicted signal peptide by SignalP v6.0 g^123^ in slow-sequential mode for eukarya were removed from our dataset. These data served as database for our proteome analysis.

For the annotation step DIAMOND v2.0.15^124^ searches against the public available databases were performed: VenomZone (de Castro, E.; Jungo, F. VenomZone. Available online: https://venomzone.expasy.org/) (downloaded on 19 January 2023), UniProtKB/Swiss-Prot Tox-Prot^40^ (downloaded on 10 January 2023), UniProtKB/Swiss-Prot v2022_05, UniProtKB/TrEMBL v2022_05^125^ (both downloaded on 20 January 2023), and annotated protein sequences from VectorBase Release 65^39,126^ (downloaded on 12 October 2023) assigned to Arthropoda. The E-value was set to a maximum of 1 × 10^−3^ in ultra-sensitive mode with all target sequences reported (--max-target-seqs 0). We then calculated the coverage of query and subject, and the similarity with the BLOSUM62^127^ matrix using BioPython v1.81^128^ for each hit. Sorting by similarity, query and subject coverage for each toxin candidate led to the resulting hit for the final analysis. With these annotations and the InterPro entries, we assigned corresponding peptide families for each putative toxin (Table S3).

#### Proteomics

For proteomics we utilized a bottom-up approach proven to be effective for venomic analyses as described earlier^128–130^. Briefly, 10 μg of lyophilized venom from 250 female individuals was dissolved in 25 mM ammonium bicarbonate with 0.6 nM ProteasMaxTM (Promega). For disulfide reduction we added 5 mM DTT for 30 min at 50 °C, followed by alkylation of free thiols via 10 mM iodacetamide for 30 min at 24 °C. After quenching the reaction by excess cysteine, trypsin was added at a 50:1 ratio and digested the venom for 16 h at 37 °C. After reaction stoppage by adding trifluoroacetic acid to a concentration of 1%, the sample was purified with C18-ZipTip (Millipore), dried them under a vacuum and redissolved the material in 10 μl of aqueous 0.1% trifluoroacetic acid.

Prior to mass spectrometry, fractionation of the venom peptides was achieved by a directly coupled chromatographic separation on a Thermo Fisher Scientific UltiMate 3000RSLCnano device. For this purpose, 1 µg of the sample material was injected into a 50 cm μPAC C18 column (Pharma Fluidics) in 0.1% formic acid at 35 °C. Peptide elution was performed using a linear gradient of acetonitrile increasing from 3% to 44% over 240 min at a flow rate of 300 nl/min. Finally, the column was washed with 72% acetonitrile. MS of the peptides was carried out on an Orbitrap Eclipse Tribrid MS (Thermo Fisher Scientific). Positive ionization with spray was established by an Advion TriVersa NanoMate (Advion BioSciences) with spray voltage set to 1.7 kV and source temperature set to 275 °C. MS scans were performed in data-independent acquisition mode with the following settings: Scanning time 3 s, mass range of *m*/*z* 375–1500 with resolution of 120,000. Auto-gain control was set to standard with a maximal injection time of 50 ms. The most intense ions occurring at each cycle with a threshold ion count of over 50,000 and charge states of 2–7 were selected with an isolation window of 1.6 *m*/*z* for higher-energy collisional dissociation (normalized collision energy 30%). MSMS spectra were acquired in the linear ion trap with rapid scan rate and normal mass range. The maximum injection time was set to 100 ms and selected precursor ions were excluded for 15 s following fragmentation.

Mass spectrometry proteomics data have been deposited in the Zenodo repository (https://zenodo.org) under the project identifier 16942242 [10.5281/zenodo.16942242].

### Matching proteomic and transcriptomic data

To identify *Ae*. *albopictus* venom compounds with proteomic coverage, the fragment spectra generated for the venom sample were matched against a database comprising a six-frame translation of the salivary gland transcriptome using the software PEAKS v. 12.5 (BSI). The analysis was performed with a parent error mass tolerance of 15 ppm and a fragment mass error tolerance of 0.1 Da. The enzyme mode was set to ‘Trypsin’ with ‘semi specific’ as digest mode. Carbamidomethylation was set as fixed post-translational modification (PTM), whereas pyro-glu from Q, amidation, half of a disulfide bridge, and carboxymethyl were considered as variable PTMs.

### Peptide identification, physicochemical properties and similarities

Physicochemical properties of salivary gland transcriptome derived cecropins were calculated using in silico tools. Briefly, we employed the antimicrobial peptide (AMP) database APD3 with its AMP calculator and predictor^131^ to find antimicrobial characteristics and similarities to known AMPs. We further employed PepCalc (Innovagen AB) to recognize basic physicochemical properties, HeliQuest^132^ to better understand the alpha-helical organization. Additionally, we used the AMP scanner vr.2^133^ to extrapolate the probability of functioning as AMP for a given sequence. Raw data from all used tools can be found in the supplementary material Table S1. Multiple sequence alignments were performed with Geneious 11.1.5 (Dormatics). Specifically, we employed ClustalW algorithm^134^ with the BLOSUM62 cost matrix^127^. Open gap costs were set to 12 and gap extension costs to 3. Further, a structural prediction with AlphaFold3^135^ was conducted to gather structural insights on the investigated peptides. ChimeraX v1.10^136^ was used for 3D structure analysis and visualizations.

### Peptide synthesis

Peptides have been synthesized by a commercial supplier of custom-made peptides (GenScript Biotech) via solid phase synthesis as described previously^137^ for further bio activity screens.

### Antimicrobial activity

For a pre-screening of antimicrobial activities, the peptides were tested against *Escherichia coli* DSMZ 102053 at a final concentration of 200 µM. Bacteria from cryo-conserved vials were transferred to tryptone soy broth (TSB) agar plates and incubated for 24 h at 37 °C. Then, single colonies were picked, transferred into 3 mL liquid TSB medium and cultivated overnight at 37 °C and 180 RPM. Subcultures were prepared in 3 mL fresh TSB medium, by adding 40 µL of overnight culture and grown for 4 h at 37 °C before testing. Optical density of each culture was measured at λ = 600 nm (OD600) using a DR2800 spectral photometer (Hach Lange) and then diluted to the start inoculum (Table S3). Peptides were available as 0.5 mg lyophilized powder, to ensure solubility stocks of 10 mmol/L in 100% DMSO (9,75 to 10,60 µL, depending on peptide molecular weight) were prepared for each peptide. They have been further diluted with TSB medium to concentrations of 400 μmol/L. As negative control 100% DMSO in a final 1:5 ratio with TSB medium was used. The 400 µmol/L peptide solution and bacteria were transferred to 96-multiwellplates in volumes of 50 µL per well each. Resulting in final volumes of 100 µL with 200 µmol/L peptide concentrations per well. Gentamicin in a final concentration of 10 µg/mL (Sigma-Aldrich) was used as positive controls. We prepared growth curves over a 48 h period at 37 °C by taking OD600 measurements every 30 min using a BioTek Eon microplate reader with short intervals of double-orbital shaking prior to each measurement. Data were analyzed and visualized using GraphPad Prism v10.0.2 (GraphPad Software) (Fig. S2).

After pre-screening, the antimicrobial effects of the synthesized peptides were further evaluated against a set of indicator strains. The used method for minimum inhibitory concentrations (MIC) determination is derived from the methodology proposed by the CLSI committee^138^. *E. coli* ATCC25922 and *Staphylococcus aureus* ATCC33592 (methicillin-resistant strain) were incubated overnight (37 °C, 180 rpm) and subsequently diluted to 5 × 10^5^ cells/mL in cation adjusted Mueller Hinton II medium (Becton Dickinson). For *E. coli* ATCC25922 an additional cell suspension was prepared in cation adjusted Mueller Hinton II medium supplemented with 100 µg/mL lipopolysaccharide (LPS) from *E. coli* 0111:B4 (Sigma-Aldrich), to investigate the potential interaction between peptide and lipopolysaccharides. As positive controls, dilution series of ceftazidime (Biomol GmbH), gentamicin (Sigma-Aldrich) ranging from 64 to 0.03 μg/mL, and ciprofloxacin (Sigma-Aldrich) ranging from 0.5 to 0.0002 µg/mL were used, to show inhibitions of all tested bacterial strains, including gram-negative, gram-positive and multidrug-resistant strains. Antibiotics stocks have been prepared in ultra-pure water as 10 mg/mL stocks and further diluted in Mueller Hinton II medium (MHII) (Tables S5 and S6). Bacterial suspensions without peptide or antibiotic control were used as negative controls. After assay incubation (37 °C, 180 rpm, 85% relative humidity), cell growth was assessed by measuring the turbidity with a microplate spectrophotometer at 600 nm (LUMIstar® Omega BMG Labtech). Growth inhibition was calculated relative to the absorption of the controls. The preculture of *Mycobacterium smegmatis* ATCC607 was incubated in brain–heart infusion broth (Becton Dickinson) for 48 h, at 37 °C and 180 RPM before the cell concentration was adjusted in Mueller Hinton II medium. Isoniazid (Sigma-Aldrich) was used at 64–0.03 µg/mL instead of gentamicin while the other two controls were done as described above. Cell viability was evaluated after 48 h (37 °C, 180 RPM, 85% relative humidity) via ATP quantification (BacTiter-Glo, Promega) according to the manufacturer’s instructions. *Candida albicans* FH2173 was incubated for 48 h at 28 °C and 180 RPM before the preculture was diluted to 1 × 10^6^ cells/mL in MHII. Tebuconazole ranging from 64 to 0.03 μg/mL and azoxystrobin at 4–0.002 µg/mL (both Cayman Chemical Company) were used as positive controls. Readout was performed via ATP quantification.

The antibacterial/antifungal MIC value is defined as the lowest concentration of an agent that inhibits the growth of a microorganism by 85% relative to the growth controls (high value). The medium background (low value) is subtracted from all measurements (AU = absorption units; luminescence for cell viability assays). The peptides stocks were dissolved in type I ultra-pure water (conductivity 0.055 µS/cm) and tested in a 12-step dilution series ranging from 50 to 0.02 μM. All concentrations were tested in triplicate. Relative growth inhibition was calculated according to:

1 $${\mathrm{Relative}}\,{\mathrm{growth}}\,{\mathrm{inhibition}}\,\left[ \% \right]=\left(1-\frac{{{AU}}_{{sample}}-{{AU}}_{{low}}}{{{AU}}_{{high}}-{{AU}}_{{low}}}\right)\times 100$$

AU = absorption units, low = medium blank, high = untreated control.

Equation (1): Calculation of relative growth.

### Hemolytic and cytotoxic activity

Erythrocytes derived from horse blood were used to detect hemolytic activities, as previously described^111^. Briefly, erythrocytes were purified by washing 300 µL of defibrinated horse blood (Thermo Fisher Scientific) with 1 mL of DPBS and centrifugation at 4 °C and 804 RCF for 5 min. Resulting supernatant was discarded and the erythrocyte containing pellet was resuspended in 1 mL of DPBS. This was repeated 3 times until the supernatant was clear. Stock solutions of cecropins were prepared at concentrations of 10 mmol/L in DMSO and mixed with the erythrocyte suspension in DPBS to gain a 1% erythrocyte suspension with 200 µmol/L of peptide. This suspension was transferred in a 96-multiwell v-bottom plate. Afterwards, the plate was incubated for 1 h at 37 °C and 130 RCF, then the plate was centrifuged at 4 °C and 804 RCF for 5 min. Finally, 50 μL of the supernatant was transferred to a flat bottom plate. Hemolytic activity was measured photometrically at λ = 405 nm (OD405) in a BioTek Eon microplate reader. We used 1% Triton X-100 as a positive control and the solvent DMSO in 1:5 ratio with DPBS as a negative control. Measurements were carried out in triplicates. Standard deviations, normalizations and visualizations were made with GraphPad Prism v10.0.2.

Cytotoxic activity of cecropins against mammalian cells was determined as previously described^139^. Briefly, Madin-Darby canine kidney II (MDCK II) cells and human-derived airway epithelial cells (Calu-3) were cultured in Dulbecco’s modified Eagle’s medium (DMEM GlutaMAX) and DMEM/F12, respectively. Both media were supplemented with 1% penicillin/streptomycin and 10% fetal calf serum (all reagents from Thermo Fisher Scientific) and the cell lines were maintained at 37 °C in a 5% CO_2_ atmosphere. Stock solutions of cecropins were prepared at concentrations of 10 mmol/L in DMSO. MDCKII cells were grown to 90% confluency in 96-well plates and treated with 200 µmol/L of the cecropins. The positive control was ionomycin (Cayman Chemicals) at 100 µM and untreated cells served as negative control. Cell viability was quantified after 48 h of incubation using the CellTiter-Glo Luminescent Cell Viability Assay (Promega). Luminescence was measured in black 96-well plates in a Synergy H4 microplate reader (Biotek). Relative light units were normalized to the untreated control set as 100% and ionomycin as 0% cell viability. Treatments with cell viability below 80% were considered toxic. Means and standard deviations were calculated from triplicate measurements using GraphPad Prism v10.0.2.

## Supplementary information

Supplementary information

Supplementary Data

## Data Availability

Raw sequencing reads are available under the project PRJEB96266 at the European Nucleotide Archive (ENA, https://www.ebi.ac.uk/ena/). All data underlying the findings are fully available and included in the manuscript. Please see Supporting Information. Mass spectrometry proteomics data have been deposited in the Zenodo repository (https://zenodo.org) under the project identifier 16942242. The bio assay raw datasets generated for this study are published as supplementary files alongside this study.
