# Supplementary material for: Vector venom: venomics of Aedes albopictus reveals a large enzyme repertoire and novel cecropins with activity against E. coli
Source: NPJ Drug Discov. 2026 Mar 2;3:7. doi: 10.1038/s44386-026-00041-w (PMC13267129; doi:10.1038/s44386-026-00041-w)
Supplement: Supplementary file 1 — Supplementary information [file 44386_2026_41_MOESM1_ESM.pdf]

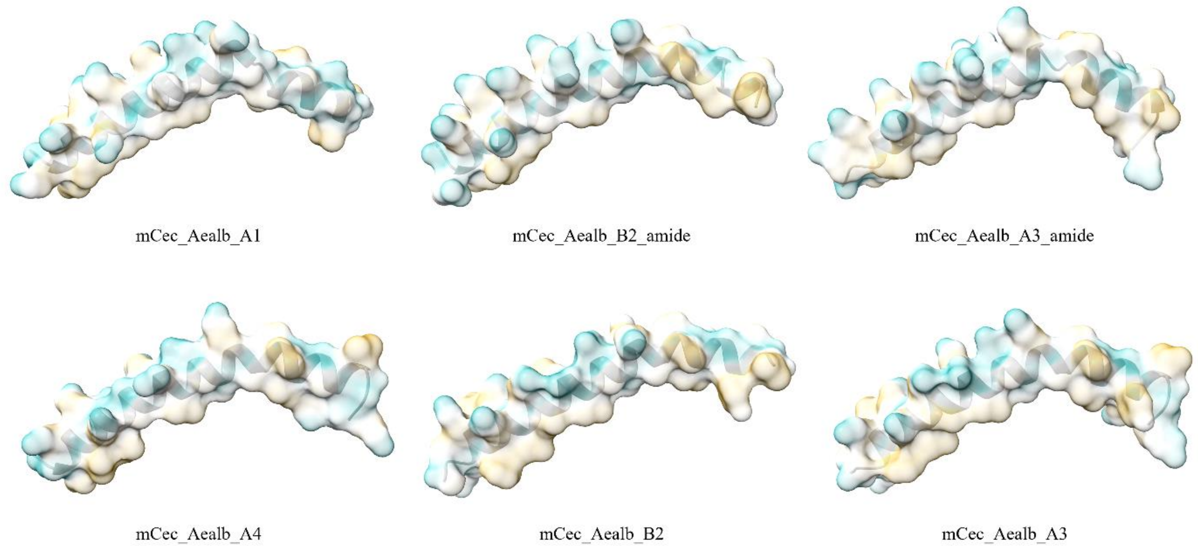

Supplementary Figure S1: 3D structure of cecropins as predicted by Alphafold3. The α-helical organization of the peptides is presented in grey. The overlayed surface view highlights hydrophobic areas in yellow and hydrophilic areas in light blue

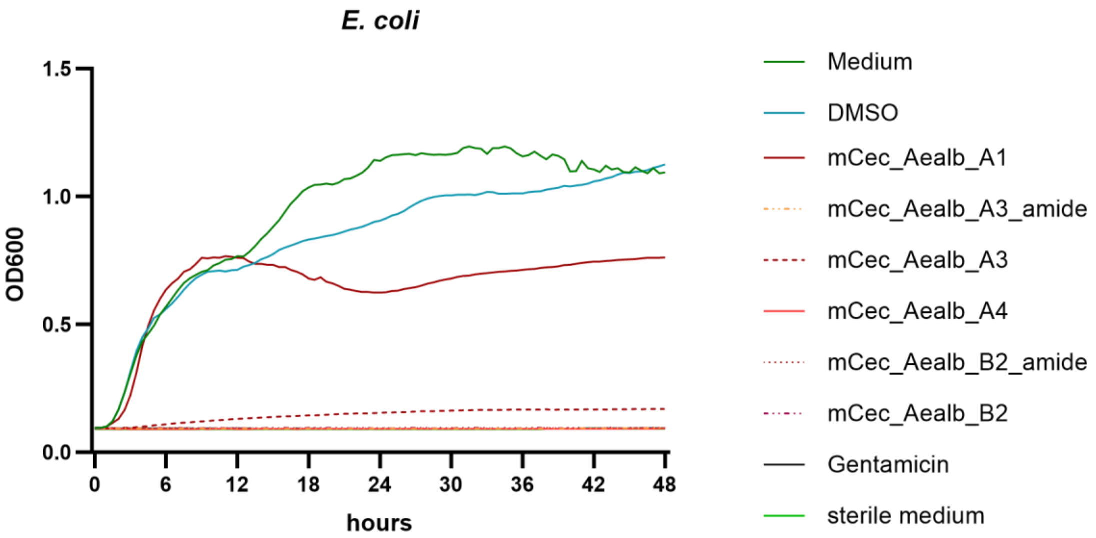

Supplementary Figure S2: Antibacterial pre-screening of cecropins. Photometrically measured 48 h growth curves of Escherichia coli DSMZ 102053, in presence of 200 µM of each cecropin. Medium shows growth of E. coli in undiluted TSB medium. Sterile medium shows the own OD of pure medium without bacterial inoculation. DMSO shows the bacterial growth in 100% DMSO diluted 1:5 with TSB medium. Gentamicin displays growth in presence of 10 µg/mL.

Supplementary Table S1: Physicochemical properties of cecropins from different tools. Antimicrobial peptide Database APD3 provides information on helical properties, hydrophobicity, hydrophobic surface, hydrophobic ratio, GRAVY index, Boman index and information’s to most similar AMPs. Heliquest gives deeper insight into the helical properties. Antimicrobial peptide scanner APS vr.2 provides general evaluation of the potential of peptides to be antimicrobial.

Name	Sequence	Helical	hydrophobic ratio	whole-residue hydrophobicity	hydrophobic surface	GRAVY	Boman idex	Most similar AMP	Organism	Similarity	Hydrophobicit	Hyd. Moment	z	Freq. Polar	Freq. NoPolar	AMP	Score
mCec_Aealb_A1	GKLKKIGKKVEKTGKHVANAAQKAGPVVAGVSALI	yes	43%	11.51	12	-0.046	0.53 kcal/mol	Cecropin C	*Aedes albopictus*	65.71%	0.190	0.389	7	0.543	0.457	yes	1.0
mCec_Aealb_A3_amide	GRLKKLGKKIEKAGKRVFNAAQKGLPVAAGVKAL	yes	44%	10.61	12	-0.247	1.11 kcal/mol	An-cecA	*Anopheles arabiensis*	85.29%	0.154	0.341	9	0.529	0.471	yes	1.0
mCec_Aealb_A3	GRLKKLGKKIEKAGKRVFNAAQKGLPVAAGVKALGR	yes	42%	11.43	12	-0.369	1.44 kcal/mol	An-cecA	*Anopheles arabiensis*	80.56%	0.117	0.318	10	0.556	0.444	yes	0.999
mCec_Aealb_A4	GGLKKLGKKLEGAGKRVFNAAEKALPVVAGAKALGK	yes	44%	11.18	10	-0.1	0.57 kcal/mol	Cecropin C	*Aedes albopictus*	97.22%	0.167	0.324	7	0.528	0.472	yes	0.999
mCec_Aealb_B2_amide	APRWKFGKKLEKVGKNVFNAAKKALPVVAGYKAL	yes	47%	7.09	9	-0.241	0.78 kcal/mol	An-cecB	*Anopheles arabiensis*	85.29%	0.274	0.270	8	0.441	0.559	yes	1.0
mCec_Aealb_B2	APRWKFGKKLEKVGKNVFNAAKKALPVVAGYKALGR	yes	44%	7.91	9	-0.364	1.12 kcal/mol	An-cecB	*Anopheles arabiensis*	80.56%	0.230	0.257	9	0.472	0.528	yes	1.0

Supplementary Table S2: Raw data for 48h E. coli growth curve

						Escherichia coli					
**time [h]**	Medium	H2O	mCec_Aealb_A1	mCec_Aealb_A3_amide	mCec_Aealb_A3	mCec_Aealb_A4	mCec_Aealb_B2_amide	mCec_Aealb_B2	Gentamicin	sterile medium
0	0,095	0,094	0,094	0,093	0,094	0,093	0,092	0,095	0,092	0,091
	0,096	0,095	0,095	0,093	0,094	0,092	0,092	0,097	0,092	0,091
	0,101	0,101	0,1	0,093	0,094	0,092	0,094	0,097	0,093	0,091
	0,12	0,121	0,114	0,093	0,094	0,092	0,095	0,093	0,093	0,091
	0,168	0,167	0,131	0,093	0,094	0,091	0,094	0,093	0,093	0,091
	0,235	0,235	0,166	0,094	0,095	0,092	0,095	0,094	0,093	0,091
	0,306	0,315	0,223	0,093	0,096	0,091	0,094	0,092	0,093	0,091
	0,378	0,395	0,312	0,093	0,098	0,091	0,095	0,093	0,093	0,091
	0,433	0,449	0,414	0,093	0,1	0,091	0,094	0,093	0,093	0,091
	0,464	0,485	0,495	0,093	0,102	0,091	0,094	0,094	0,093	0,091
	0,496	0,525	0,556	0,093	0,105	0,091	0,094	0,092	0,093	0,091
	0,539	0,539	0,601	0,093	0,107	0,091	0,094	0,096	0,093	0,091
6	0,57	0,561	0,635	0,093	0,109	0,091	0,094	0,092	0,093	0,091
	0,602	0,582	0,66	0,093	0,112	0,091	0,095	0,093	0,093	0,091
	0,634	0,608	0,679	0,093	0,114	0,091	0,094	0,095	0,093	0,091
	0,662	0,636	0,706	0,093	0,115	0,091	0,095	0,094	0,093	0,091
	0,681	0,66	0,716	0,093	0,117	0,091	0,094	0,093	0,093	0,091
	0,693	0,681	0,736	0,093	0,119	0,091	0,094	0,092	0,092	0,091
	0,706	0,694	0,76	0,093	0,121	0,092	0,094	0,094	0,093	0,091
	0,712	0,706	0,758	0,093	0,122	0,091	0,095	0,095	0,093	0,091
	0,73	0,708	0,762	0,093	0,124	0,092	0,094	0,092	0,093	0,091
	0,739	0,71	0,761	0,093	0,126	0,092	0,094	0,096	0,092	0,091
	0,753	0,707	0,767	0,093	0,127	0,092	0,094	0,094	0,093	0,091
	0,756	0,712	0,764	0,093	0,129	0,092	0,095	0,094	0,093	0,091
12	0,767	0,713	0,76	0,093	0,13	0,091	0,094	0,095	0,093	0,091
	0,766	0,726	0,759	0,093	0,132	0,091	0,094	0,094	0,093	0,091
	0,783	0,732	0,746	0,093	0,133	0,091	0,094	0,092	0,093	0,091
	0,807	0,741	0,737	0,093	0,134	0,092	0,094	0,093	0,092	0,091
	0,833	0,754	0,737	0,093	0,135	0,092	0,094	0,093	0,092	0,091
	0,856	0,763	0,733	0,093	0,137	0,092	0,094	0,096	0,092	0,091
	0,88	0,774	0,733	0,093	0,138	0,092	0,093	0,095	0,092	0,091
	0,907	0,79	0,724	0,093	0,139	0,092	0,093	0,094	0,092	0,091
	0,941	0,801	0,724	0,093	0,14	0,092	0,095	0,095	0,092	0,091
	0,972	0,808	0,713	0,093	0,141	0,092	0,093	0,095	0,092	0,091
	0,995	0,817	0,705	0,093	0,142	0,091	0,093	0,094	0,093	0,091
	1,02	0,826	0,696	0,093	0,143	0,092	0,093	0,094	0,092	0,091
18	1,036	0,832	0,679	0,093	0,144	0,092	0,094	0,095	0,092	0,091
	1,046	0,836	0,674	0,093	0,145	0,092	0,094	0,096	0,093	0,091
	1,047	0,84	0,684	0,093	0,146	0,092	0,093	0,095	0,092	0,091
	1,05	0,845	0,667	0,093	0,147	0,092	0,094	0,095	0,092	0,091
	1,047	0,849	0,661	0,093	0,148	0,092	0,093	0,095	0,092	0,091
	1,054	0,856	0,651	0,093	0,149	0,092	0,094	0,095	0,092	0,091
	1,068	0,863	0,639	0,093	0,15	0,092	0,093	0,095	0,092	0,091
	1,07	0,87	0,635	0,093	0,15	0,092	0,093	0,094	0,092	0,091
	1,081	0,876	0,631	0,093	0,152	0,092	0,094	0,096	0,092	0,091
	1,093	0,882	0,627	0,093	0,152	0,092	0,093	0,095	0,092	0,091
	1,113	0,891	0,627	0,093	0,152	0,092	0,093	0,096	0,092	0,091
	1,143	0,9	0,624	0,093	0,153	0,092	0,094	0,093	0,092	0,091
24	1,14	0,905	0,624	0,093	0,154	0,092	0,094	0,093	0,092	0,091
	1,153	0,914	0,625	0,093	0,155	0,092	0,094	0,094	0,092	0,091
	1,162	0,925	0,63	0,093	0,156	0,092	0,094	0,093	0,092	0,091
	1,163	0,932	0,632	0,093	0,157	0,092	0,094	0,094	0,092	0,091
	1,165	0,943	0,638	0,093	0,157	0,092	0,094	0,095	0,092	0,091
	1,167	0,958	0,642	0,093	0,158	0,092	0,093	0,096	0,092	0,091
	1,162	0,972	0,646	0,093	0,159	0,092	0,094	0,096	0,092	0,091
	1,17	0,983	0,652	0,093	0,16	0,092	0,094	0,096	0,092	0,091
	1,166	0,992	0,659	0,093	0,16	0,092	0,093	0,095	0,092	0,091
	1,163	0,998	0,664	0,093	0,161	0,092	0,094	0,093	0,092	0,091
	1,164	1,002	0,671	0,093	0,162	0,092	0,095	0,093	0,092	0,091
	1,163	1,004	0,675	0,093	0,162	0,092	0,094	0,093	0,092	0,091
30	1,166	1,005	0,679	0,093	0,163	0,092	0,095	0,093	0,092	0,091
	1,171	1,007	0,686	0,093	0,163	0,092	0,094	0,095	0,092	0,091
	1,189	1,007	0,689	0,093	0,164	0,092	0,093	0,096	0,092	0,091
	1,195	1,008	0,691	0,093	0,164	0,092	0,094	0,096	0,092	0,091
	1,19	1,006	0,695	0,093	0,165	0,092	0,094	0,096	0,092	0,091
	1,187	1,01	0,698	0,093	0,165	0,092	0,094	0,096	0,092	0,091
	1,167	1,018	0,7	0,093	0,166	0,092	0,093	0,096	0,092	0,091
	1,189	1,017	0,703	0,093	0,166	0,092	0,094	0,092	0,092	0,091
	1,19	1,01	0,706	0,093	0,166	0,092	0,095	0,092	0,092	0,091
	1,195	1,011	0,707	0,093	0,166	0,092	0,093	0,096	0,092	0,091
	1,188	1,012	0,709	0,093	0,167	0,092	0,094	0,093	0,092	0,091
	1,17	1,012	0,71	0,093	0,167	0,092	0,095	0,093	0,092	0,091
36	1,157	1,012	0,713	0,093	0,167	0,092	0,094	0,094	0,092	0,091
	1,162	1,017	0,715	0,093	0,167	0,092	0,094	0,094	0,092	0,091
	1,175	1,019	0,717	0,093	0,167	0,092	0,095	0,096	0,092	0,091
	1,159	1,021	0,72	0,093	0,167	0,092	0,094	0,096	0,092	0,091
	1,146	1,027	0,723	0,094	0,168	0,092	0,095	0,096	0,092	0,092
	1,164	1,031	0,724	0,093	0,166	0,092	0,094	0,094	0,092	0,092
	1,16	1,035	0,727	0,093	0,167	0,092	0,094	0,094	0,092	0,092
	1,145	1,042	0,732	0,093	0,167	0,092	0,095	0,094	0,092	0,092
	1,098	1,039	0,733	0,093	0,167	0,092	0,094	0,095	0,092	0,092
	1,099	1,043	0,737	0,093	0,167	0,092	0,095	0,093	0,092	0,092
	1,139	1,046	0,74	0,094	0,167	0,092	0,095	0,092	0,093	0,093
	1,11	1,05	0,743	0,093	0,168	0,092	0,094	0,092	0,093	0,093
42	1,106	1,059	0,745	0,094	0,167	0,092	0,095	0,092	0,093	0,093
	1,095	1,063	0,746	0,094	0,168	0,092	0,094	0,092	0,093	0,093
	1,12	1,071	0,748	0,093	0,167	0,092	0,095	0,096	0,093	0,093
	1,105	1,076	0,749	0,094	0,168	0,092	0,094	0,097	0,093	0,093
	1,11	1,085	0,752	0,094	0,168	0,092	0,094	0,094	0,093	0,093
	1,096	1,093	0,753	0,094	0,168	0,093	0,094	0,094	0,093	0,093
	1,094	1,092	0,754	0,094	0,168	0,092	0,095	0,093	0,093	0,093
	1,114	1,097	0,756	0,094	0,169	0,093	0,096	0,093	0,094	0,092
	1,101	1,098	0,758	0,094	0,168	0,092	0,095	0,094	0,094	0,092
	1,09	1,103	0,761	0,094	0,169	0,092	0,094	0,095	0,094	0,092
	1,11	1,112	0,761	0,094	0,169	0,092	0,095	0,095	0,094	0,092
	1,09	1,118	0,761	0,095	0,169	0,093	0,095	0,095	0,094	0,092
48	1,094	1,126	0,762	0,094	0,169	0,093	0,096	0,094	0,094	0,092

Supplementary **Table S3**: Startinoculum for 48h E. coli growth curve.

**Bacteria**	**DSMZ**	**Start inoculum (OD600)**	**Medium**
E. coli	102053	0.000312	tryptone soy broth (TSB)

Supplementary Table S4: Minimal Inhibitory concentration assay results

**tested cpd stock (5mM in water) n=3**	**MIC (µM)**					
	Ec		***Ms***	***Sa***		***Ca***
	**ATCC 25922**		**ATCC607**	**ATCC 33592**		**FH2173**
				**MRSA**		
	**MHII**	**+LPS**	**MHII**	**MHII**		**MHII**
mCec_Aealb_A1	50	nd	>50	>50	>50	25
mCec_Aealb_A3_amide	0.2	3.125	>50	>50	50	25
mCec_Aealb_A3	0.4	6.25	>50	>50	>50	25
mCec_Aealb_A4	6.25-3.125	6.25	>50	>50	50	25
mCec_Aealb_B2_amide	0.4	6.25	25-12.5	>50	50	50
mCec_Aealb_B2	0.4	12.5	>50	>50	>50	50

**MIC (µg/mL)**						
CFZ	0.5-0.25	0,5	>64	>64	TEB	0,125
CIP	0,008	0.016-0.008	0.5-0.25	0.5-0.25	AZO	0,6
GEN/INZ*	1-0.25	1	*2	0.25-0.125		

Ec	Escherichia coli
Ms	Mycobacterium smegmatis
Sa	Staphylococcus aureus
Af	Aspergillus aureus
Ca	Candida albicans
MHII	cation-adjusted Mueller Hinton 2 Medium
CFZ	ceftazidime
CIP	ciprofloxacin
GEN	gentamicin
INZ	isonazid
TEB	tebuconazole
AZO	azoxystrobin
NYS	nystatin

Supplementary Table S5: Generic plate design for MIC assy used to test 1 peptide against 1 microorganis

	1	2	3	4	5	6	7	8	9	10	11	12	
A	1µL vehicle	1:2	1:2	1:2	1:2	1:2	1:2	1:2	1:2	1:2	1:2	1:2	bacterial/yeast solution with vehicle control (water); supplemented vehicle volume is equivalent to the volume in the peptide test wells; A1 = 1µL followed by 1:2 dilution to A12
B	1µL sample A	1:2	1:2	1:2	1:2	1:2	1:2	1:2	1:2	1:2	1:2	1:2	bacterial/yeast solution with concentrated sample stock solution B1, C1, D1 = 1µL followed by 1:2 dilution to column 12
C	1µL sample B	1:2	1:2	1:2	1:2	1:2	1:2	1:2	1:2	1:2	1:2	1:2	
D	1µL sample C	1:2	1:2	1:2	1:2	1:2	1:2	1:2	1:2	1:2	1:2	1:2	
E	1 µL ABx1	1:2	1:2	1:2	1:2	1:2	1:2	1:2	1:2	1:2	1:2	1:2	Antibiotic standard 1 to validate the assay. Tested concentration range is specified in Table SXYZ. 1 µL of a 100fold concentrated stock solution is supplemented and consequtitly diluted 1:2
F	1 µL ABx2	1:2	1:2	1:2	1:2	1:2	1:2	1:2	1:2	1:2	1:2	1:2	Antibiotic standard 2 to validate the assay. Tested concentration range is specified in Table SXYZ. 1 µL of a 100fold concentrated stock solution is supplemented and consequtitly diluted 1:2
G	1 µL ABx3	1:2	1:2	1:2	1:2	1:2	1:2	1:2	1:2	1:2	1:2	1:2	Antibiotic standards 3 to validate the assay. Tested concentration range is specified in Table SXYZ. 1 µL of a 100fold concentrated stock solution is supplemented and consequtitly diluted 1:2
H	MED	MED	MED	MED	MED	BY	BY	BY	BY	BY	BY	BY	Medium background measurement; bacterial/yeast suspension to compare with speficially dilution series of vehicle

Supplementary Table S6: Antibiotic standards for MIC assay

	Antibiotic Standards: ranges tested in 12 point dilution series [µg/mL]					
	Ceftazidime	Ciprofloxacin	Gentamicin	Isoniazid	Tebuconazole	Azoxystrobin
E.coli ATCC25922	64 - 0.03	0.5- 0.0002	64 - 0.03			
S. aureus ATCC33592	64 - 0.03	0.5- 0.0002	64 - 0.03			
M.smegmatis ATCC607	64 - 0.03	0.5- 0.0002		64 - 0.03		
C. albicans FH2173					64 - 0.03	4 - 0.02

Supplementary Table S7: Raw reads for cytotoxic activity tests

MDCKII			
Untreated	18298	17144,67	18674,67
mCec_Aealb_A1	18559,33	19971,67	18683
mCec_Aealb_B2_amide	6133,667	5843,667	5661,333
mCec_Aealb_A3_amide	17515,33	17653	17054,67
mCec_Aealb_A4	20485,67	20698,33	20459,33
mCec_Aealb_B2	13677,33	13087	12502,33
mCec_Aealb_A3	18712	17919	17481
Ionomycin	8,333	6,333	9

Calu-3			
Untreated	34277,33	33770,33	34716,67
mCec_Aealb_A1	33311,67	33025,33	34754,67
mCec_Aealb_B2_amide	8968	9984,667	11407,67
mCec_Aealb_A3_amide	33966,33	33022,67	33366
mCec_Aealb_A4	33251,33	32551	33001,67
mCec_Aealb_B2	32703,67	33062,67	33394
mCec_Aealb_A3	29393,33	34440	34343,67
Ionomycin	111,667	122,333	160

Supplementary Table S8: Raw reads for hemolytic activity tests

Triton X 100	0,799	0,799	0,798
Triton X 100	0,753	0,752	0,752
Triton X 100	0,716	0,715	0,715
mCec_Aealb_A1	0,064	0,065	0,065
mCec_Aealb_A1	0,06	0,061	0,061
mCec_Aealb_A1	0,069	0,069	0,069
mCec_Aealb_B2_amide	0,091	0,09	0,09
mCec_Aealb_B2_amide	0,088	0,088	0,088
mCec_Aealb_B2_amide	0,077	0,077	0,077
mCec_Aealb_A3_amide	0,06	0,059	0,06
mCec_Aealb_A3_amide	0,062	0,062	0,062
mCec_Aealb_A3_amide	0,063	0,063	0,063
mCec_Aealb_A4	0,066	0,066	0,066
mCec_Aealb_A4	0,059	0,06	0,059
mCec_Aealb_A4	0,062	0,062	0,062
mCec_Aealb_B2	0,065	0,065	0,065
mCec_Aealb_B2	0,067	0,067	0,067
mCec_Aealb_B2	0,105	0,105	0,105
mCec_Aealb_A3	0,06	0,06	0,06
mCec_Aealb_A3	0,069	0,069	0,069
mCec_Aealb_A3	0,06	0,06	0,06
ddH2O	0,061	0,061	0,061
ddH2O	0,067	0,067	0,067
ddH2O	0,064	0,064	0,064
A. mellifera 50µg/mL	0,621	0,62	0,619
A. mellifera 50µg/mL	0,646	0,645	0,644
A. mellifera 50µg/mL	0,584	0,583	0,582
DMSO	0,071	0,071	0,071
DMSO	0,07	0,07	0,07
DMSO	0,069	0,069	0,069

Supplementary Data: The table labeled as supplementary data includes the transcriptomic and proteomic data after manual annotation.
